# Genome-wide association study of lipase and esterase in wholegrain wheat flour (*Triticum aestivum* L.)

**DOI:** 10.1371/journal.pone.0282510

**Published:** 2023-03-09

**Authors:** Chun Yue Wei, Steven Yates, Dan Zhu, Andreas Hund, Bruno Studer, Laura Nyström

**Affiliations:** 1 Laboratory of Food Biochemistry, Institute of Food, Nutrition and Health, ETH Zurich, Zurich, Switzerland; 2 Molecular Plant Breeding, Institute of Agricultural Sciences, ETH Zurich, Zurich, Switzerland; 3 Crop Science, Institute of Agricultural Sciences, ETH Zurich, Zurich, Switzerland; Universidad Nacional Autonoma de Mexico Centro de Nanociencias y Nanotecnologia, MEXICO

## Abstract

Lipase activity is one of the main causes of the lipid rancidity in wholegrain wheat flour, leading to its short shelf life. Genetically diverse wheat germplasm offers potential for the selection of wheat cultivars with low lipase activity for stable wholegrain end use. This study evaluated 300 European wheat cultivars harvested in 2015 and 2016 on the genetic association of lipase and esterase activities in wholegrain wheat flour. Esterase and lipase activities in wholegrain flour were measured photometrically with *p*-nitrophenyl butyrate and *p*-nitrophenyl palmitate as substrates, respectively. Both enzyme activities showed wide ranges among all cultivars within each year, with differences up to 2.5-fold. The two years showed low correlations between each other, indicating a large environmental impact on the enzyme activities. Cultivars ‘Julius’ and ‘Bueno’ were suggested to be better suited for stable wholegrain products, as they had consistently low esterase and lipase activities compared to the other cultivars. A genome-wide association study revealed associations with single nucleotide polymorphisms in genes located on the high-quality wheat genome sequence of the International Wheat Genome Sequencing Consortium. Eight and four candidate genes were tentatively proposed to be associated to esterase and lipase activity, respectively, in wholegrain flour. Our work shows esterase and lipase activities from a new perspective, that combines reverse genetics to understand the underlying causes. This study outlines the possibilities and limitations to improve lipid stability of wholegrain wheat by genomics-assisted breeding methods, thereby offering new opportunities to optimize the quality of wholegrain wheat flour and wholegrain products.

## Introduction

Whole grains are known for their health benefits of reducing the risks of cardiovascular disease, coronary heart disease, and type II diabetes (European Commission, 2020). However, wholegrain wheat flour and products are facing a serious quality challenge of short shelf life caused by low lipid stability [1]. The shelf life of wholegrain flour is only 3–6 months, which is less than half of white flour (up to one year). Lipid rancidity and oxidation in wholegrain flour lead to a reduction of its sensory acceptance (unpleasant smell and taste), nutritional value, and technological functionalities. One of the major causes of the low lipid stability in wholegrain flour is considered to be the hydrolytic enzymes from the bran and germ fractions, lipase and esterase [1].

Lipase (EC 3.1.1.3) and other esterases hydrolyze the ester bonds of lipids and release free fatty acids, increasing their susceptible to oxidation. The activities of these hydrolytic enzymes in wholegrain wheat flour were observed by the accumulation of free fatty acids after eight weeks of storage at 26°C [2]. With *p-*nitrophenyl palmitate and *p-*nitrophenyl butyrate as substrates, lipase and esterase activities in defatted wholegrain wheat flour from six wheat varieties were reported to vary from 30–580 and 650–2500 U/kg, respectively [3]. To improve the stability of the wholegrain flours, attempts have been made to inhibit the enzyme activities during processing, including steaming [4, 5], microwave treatment [6], and gamma radiation [7]. These treatments, however, are not only energy consuming, but may also induce quality change and decrease the nutritional value of the wholegrain product.

Alternatively, numerous wheat germplasm resources with large diversity [8] suggest the possibility of cultivar selection with low enzyme activities. A three-fold variance of lipase and esterase activities was observed among 66 wheat cultivars in our previous study [9]. In addition, genetic dependency of lipase and esterase activities was reported with the broad-sense heritability of 0.4 and 0.7, respectively [9]. Cubadda, Bozzini [10] located the correlations with several esterase isozymes on the wheat chromosomes 3A, 3B, and 3D. More recently, gene for a GDSL-like lipase (having an amino acid sequence of Gly (G), Asp (D), Ser (S) and Leu (L) found around the active site Ser (S) in serine-lipase) was mapped on the short arm of chromosome 7D in wheat [11, 12]. Due to the genetic impact on the enzyme activities, it is viable to breed wheat varieties with low lipase and esterase activities. However, phenotyping of the enzyme activities in wheat flour is laborious and low throughput. It is challenging to target these traits by traditional breeding. Nevertheless, marker-assisted breeding helps to select appropriate recombination events and speed up the selection process. To develop genetic markers, genes responsible for the esterase and lipase activities need to be identified and located on the genome.

Quantitative trait locus (QTL) analysis reveals the association of genetic markers with a trait of interest, such as the enzyme activities. With the advancement in genotyping, a high number of markers in the wheat genome are available, which enables genome-wide association studies (GWAS). GWAS is a promising approach to map genome regions associated with the traits. A large set of over 300 wheat lines (GABI-panel) suitable for GWAS was established by the German Plant Genome Research Program [13]. The genotype data including 90,000 gene-associated and genome-wide distributed single nucleotide polymorphism (SNP) markers of the GABI-panel has been collected [14]. Studies with the GABI-panel has mapped QTL controlling plant height [15], temperature response [16], stomatal density [17], heading date [18], senescence behavior [19], thousand grain weight [20], and fungal infection [21].

The aim of this study is first to investigate the variation of esterase and lipase activities in contemporary wheat germplasm, and to evaluate the cultivars on the suitability for stable wholegrain use. Secondly, we aim to identify QTL associated with esterase and lipase to provide genetic markers for selection. Finally, genes controlling the activities of the enzymes were proposed. The results provide valuable information for breeding of low-esterase/lipase-activity wheat cultivars. This, on the other hand, will allow the food industry and consumers to benefit from the stable wholegrain flour and products with prolonged shelf life.

## Materials and methods

### Samples and chemicals

A total of 300 wheat cultivars from the GABI-panel [13] was evaluated for esterase and lipase activities. The wheat cultivars were grown in the field imaging platform (FIP) [22] at the field station of ETH Zurich in Lindau‐Eschikon (47.449° N, 8.682° E, 520 m above sea level; soil type: Gleyic Cambisol) in Switzerland over two years, 2015 and 2016. The cultivars were grown in microplots of 1.2 m × 1.7 m size with a sowing density of 400 m^−1^ in two lots [16]. For all cultivars, wheat kernels harvested from one of the two replicates were randomly selected for analysis. The samples were milled to wholegrain flour with a 0.5 mm sieve (ZM200; Retsch, Haan, Germany) and stored at −20 °C until analysis. A commercial wheat grain (Egli bio Reform AG, Demeter, Switzerland) was used as an in‐house reference. It was milled and analyzed identically as the other samples and its enzyme activities were measured daily in triplicate.

All chemicals were purchased from Merck (Darmstadt, Germany), except for *p*‐nitrophenyl palmitate, which was purchased from Alfa Aesar (Heysham, UK). Commercial lipase *Candida antarctica* A (NS‐40010) was purchased from Novozymes (Bagsvaerd, Denmark).

### Enzyme activities and lipid content

The extraction of the enzymes and the activity measurement were carried out as described by Wei et al. [9]. Briefly, *p*‐nitrophenyl butyrate and *p*‐nitrophenyl palmitate were used as the substrates for esterase and lipase, respectively. The slope of absorption change at 405 nm from 5 to 15 min were used to calculate the activity. One unit (1 U = 16.7 nkat) was defined as the amount of enzyme that generates of 1 μmol *p*-nitrophenol per minute at 37 °C and pH 8.0. Commercial lipase *Candida antarctica* (4880 U/kg with p-nitrophenyl palmitate as substrate) and the in‐house reference sample (22 U/kg with *p-*nitrophenyl palmitate as substrate) were used to test the reliability and repeatability of the method.

The non-starch lipid contents in the wheat flour were measured according to Wei et al. [9] with an accelerated solvent extractor (ASE 350; Dionex, Sunnyvale, CA, USA) using petroleum ether (bp 40–60°C) for extraction.

### Association analysis

The association of the enzyme activities and the genotypic data [14] were analyzed in PLINK [23] as described by Yates et al. [17]. Haplotype mapping was performed with a sliding window approach of three consecutive SNPs and a linear regression model. No co-factors were used to correct for population structure, because there is no clear population structure in this population (Kollers et al. 2013). For the identification of significantly associated markers, Bonferroni correction was applied by multiplying the 0.05 *p* significance with the number of SNP makers. The genome information was retrieved from the International Wheat Genome Sequencing Consortium (IWGSC) database [24]. After the significant QTL was located on the chromosome, genes that might encode lipase or esterase were searched within the interval of the marker position plus and minus 1 M base pair.

Since trait associations were found between several neighbouring markers, we calculated the pairwise *R*^*2*^ (a measure of linkage disequilibrium, using the R package “genetics” v.1.3.8.1.3) between markers. Markers were grouped together into linkage disequilibrium blocks when the pairwise *R*^*2*^ was greater than 0.9. From these data we found 150 unique linkage disequilibrium blocks that had a mean size of 8,783,545 bp and median of 2,191,028 bp. Thus, our search of one million base-pairs up and downstream of the QTL peaks is conservative.

### Statistical analysis

All results were reported as mean plus and minus standard deviation of technical triplicates. Software R (version 3.6.3) was used for statistical analysis. Two-way ANOVA was performed by IBM SPSS Statistics 24 with cultivar, years, and the interaction between cultivar and years as factors.

## Results and discussion

**Fig 1:**
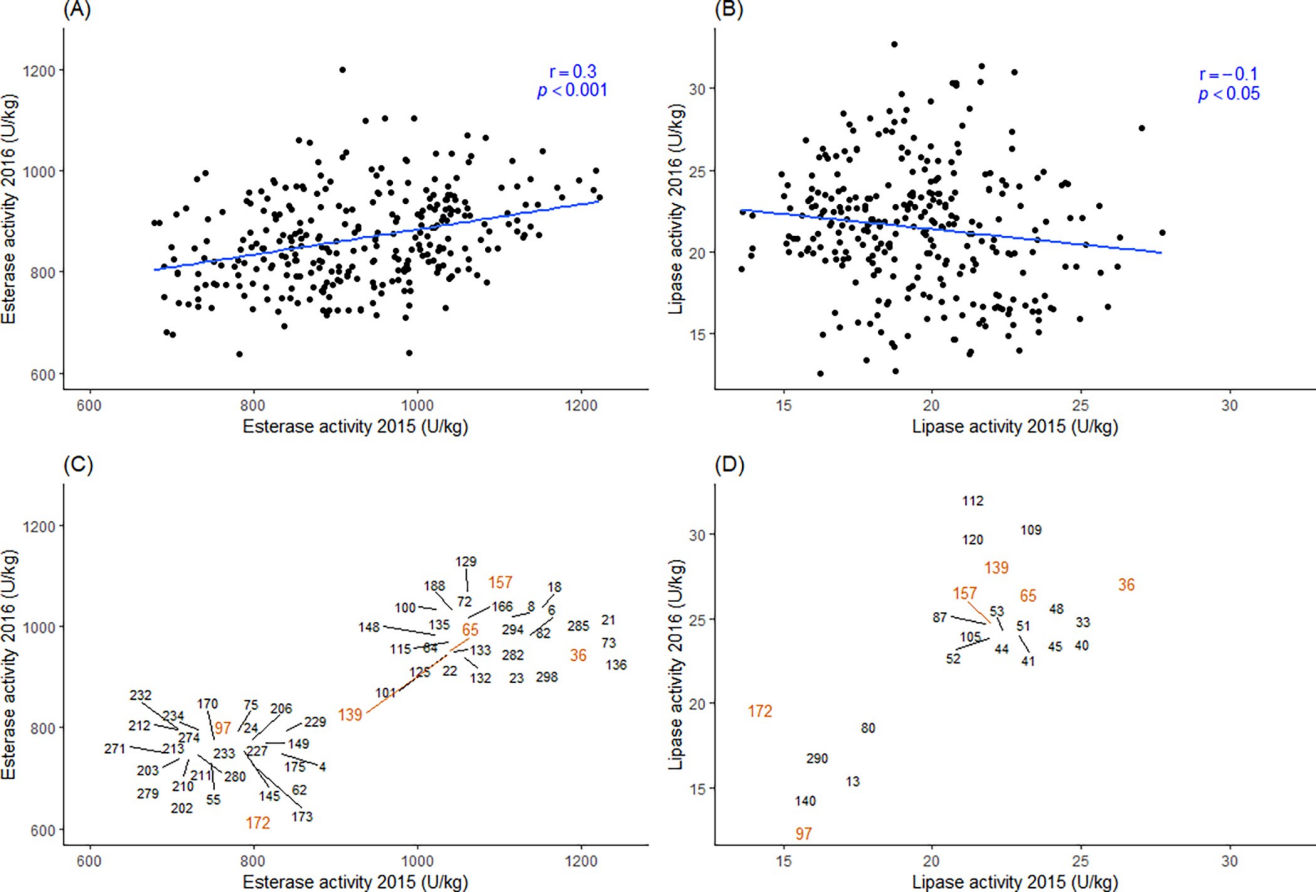
Esterase (A) and lipase (B) activities of 300 wheat cultivars grown in 2015 and 2016. Each dot and number represent one wheat cultivar. Linear regression model of esterase activity from the two years is represented by the blue line. Cultivars with their esterase (C) and lipase (D) activities in the upper and lower quantiles in both years. Cultivars overlapped in (C) and (D) were labeled in orange.

Esterase and lipase activities of 300 wheat cultivars harvested in 2015 and 2016 were analyzed (Table 1, Fig 1, S1 Table). The two enzyme activities present a weak positive significant correlation (*r* = 0.4, *p* < 0.001). A two-way ANOVA showed highly significant differences (*p* < 0.001) in enzyme activities for the factors, cultivar, years, and the interaction between cultivar and year. The variation between the cultivars was demonstrated by an up to 2.5-fold difference among the cultivars of each enzyme within one year (Table 1). The lowest esterase activity was observed in cultivar 172 ‘Bueno’ grown in 2016 with 638 ± 39 U/kg. ‘Bueno’ also had the lowest lipase activity (13.6 ± 1.3 U/g) in 2015. Cultivar 97 ‘Julius’ grown in 2016 showed the lowest lipase activity of 12.6 ± 0.5 U/kg. It was reported that ‘Julius’ had a low esterase best linear unbiased prediction (BLUP) value [9]. This indicates that the cultivars ‘Bueno’ and ‘Julius’ may have high stability as wholegrain flour because of their low esterase and lipase activities. The highest esterase and lipase activities were observed in cultivar 136 ‘Intact’ from 2015 and cultivar 114 ‘Bastide’ from 2016, with the value of 1’223 ± 44 U/kg and 27.7 ± 0.8 U/kg, respectively. Wholegrain flour made of wheat cultivars with high esterase and lipase activities, such as ‘Intact’ and ‘Bastide’, is expected to have short shelf life due to potential lipid rancidity. Though the evaluation of lipid oxidation in food products with low lipid and low moisture contents as well as low water activity (a_w_) is challenging and does not always follow the classical pattern of hydroperoxide formation and degradation to secondary oxidation products, the cultivar Julius showed also comparably slow formation of the primary oxidation products measured with peroxide value, despite the low levels of antioxidants like tocopherols [25]. Therefore, one can hypothesize that the low levels of endogenous antioxidants can be compensated with a low lipase and esterase activities in improving the stability of wholegrain wheat flours.

**Table 1:** The minimum, maximum, and mean values of esterase and lipase activities measured in 300 wheat cultivars grown in 2015 and 2016. Unit: U/kg.

	Esterase								Lipase							
	2015				2016				2015				2016			
Minimum	679	±	52	**AUTAN**	638	±	39	**BUENO**	13.6	±	1.3	**BUENO**	12.6	±	0.5	**JULIUS**
Maximum	1,223	±	44	**INTACT**	1,200	±	64	**MARSHAL**	27.7	±	0.8	**ACTROS**	32.7	±	2.0	**BASTIDE**
Mean	926				866				19.5				21.5			

All the wheat cultivars were collected from the two growing seasons 2015 and 2016. A weak significant correlation (r = 0.3, *p* < 0.001) was observed between the two years for esterase activity (Fig 1A). However, a minor negative correlation (r = -0.1, *p* = 0.02) was observed for lipase activity (Fig 1B), although in our earlier study the lipase heritability was estimated to be 0.4 [9]. The weather conditions in the two years were very different: the year 2016 had an exceptionally wet summer, which resulted in a historic yield reduction and disease pressure [26] and distinctly different senescence behavior [19]. The weak correlation between the two years suggests that lipase activity is strongly influenced by environmental conditions. Hence a better understanding would be needed what is driving the changes in lipase activity.

Nevertheless, some cultivars were more resistant to different environments over the two grown years. Cultivars belong to the lower and upper quantiles of esterase (Fig 1C) and lipase (Fig 1D) activities in both years are illustrated, among which some cultivars are consistent for both activities. Cultivars 97 ‘Julius’ and 172 ‘Bueno’ were in the lower quantile of both esterase and lipase in both years. Cultivars 36 ‘Drifter’, 65 ‘Paroli’, 139 ‘Kleber’, and 157 ‘Raison’ were in the upper quantile of both enzymes in both years. These cultivars were less influenced by environment conditions. Cultivar ‘Julius’ and ‘Bueno’ demonstrated low enzyme activity and high consistency in different environments, and thus they are potentially suitable for stable wholegrain use with prolonged shelf life.

Lipid contents of the cultivars grown in 2016 showed a 1.5-fold variance (S1 Table). Cultivar 229 ‘SATYNA’ (1.13% ± 0.10) and 200 ‘Eriwan’ (1.86% ± 0.05) had the lowest and highest lipid contents, respectively. Comparison of the experimental means of lipid content and esterase activity showed a weak correlation (r = 0.27, *p* < 0.001), and no correlation between lipase activity.

**Fig 2:**
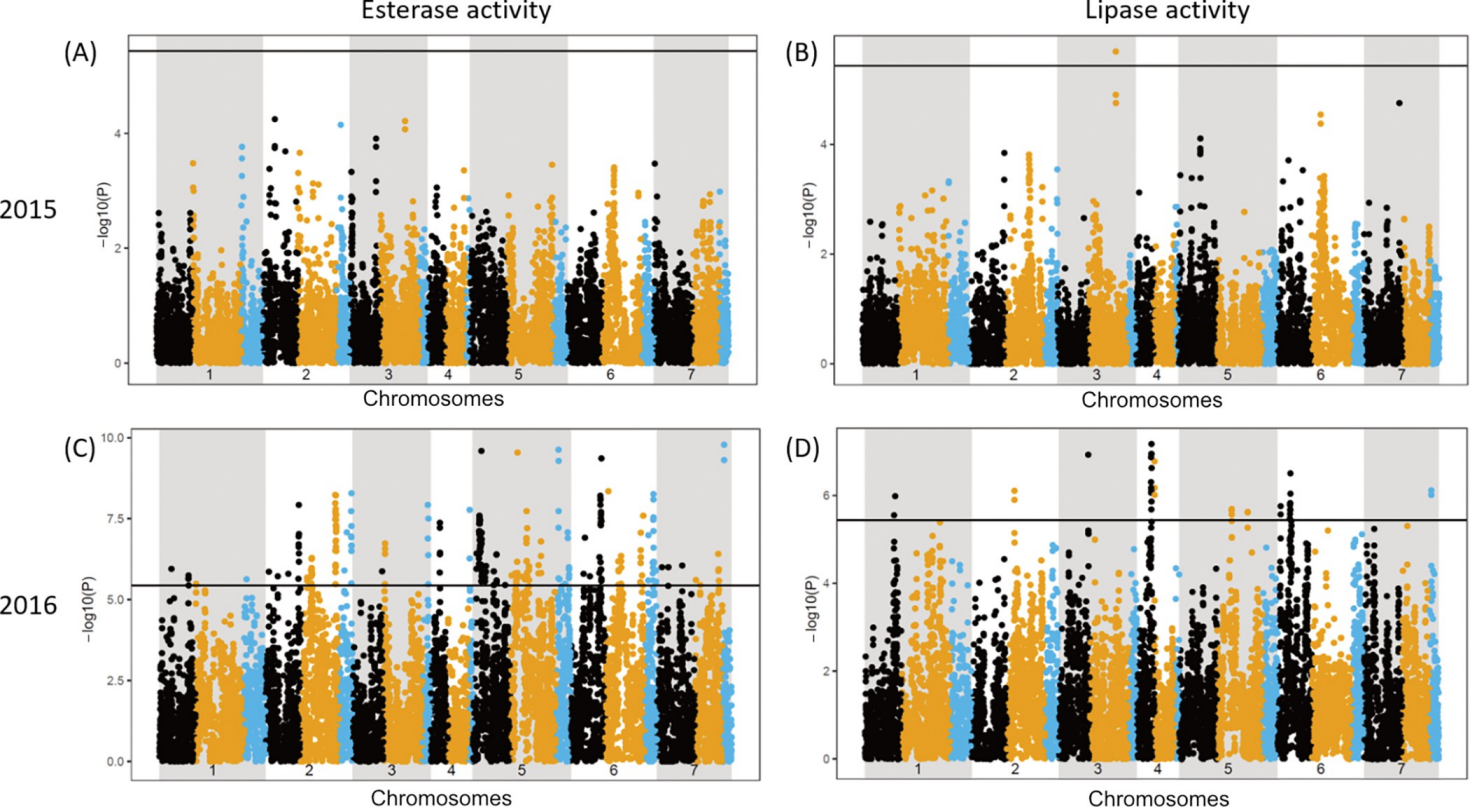
Manhattan plots for esterase (A, C) and lipase (B, D) activity of the GABI-panel harvested in 2015 (A, B) and 2016 (C, D). The x-axis shows the relative position across 7 chromosomes and the y-axis the -log_10_P-value. The three genomes, A, B and D, are represented by black, orange and blue, respectively. Significant line is drawn at FDR p = 0.02.

To identify genomic regions associated with esterase and lipase activities, the SNP markers of the GABI-panel [14] were used for haplotype mapping with a sliding window of three consecutive markers. Significant markers were observed for both esterase and lipase in 2016, but only one SNP was significantly associated with lipase in 2015 (Fig 2, S2 Table). The two years did not show any overlapping significant markers, as expected from the low correlations. Esterase and lipase activities measured in 2016 had more significant associations. In the regions of 1 M base pairs above and below the most left of the three markers used for the sliding window approach, the sequences were BLAST searched against the wheat genome from the IWGSC database [24]. Genes that might underpin esterase or lipase activities were selected on the basis of their functional annotation (Table 2). Genes putatively involved in esterase activity were located on chromosome 2A, 2B, and 2D, and genes putatively involved in lipase activity were on chromosome 3B, 4A, and 6D. The results complement the previously reported GDSL-like lipase gene on chromosome 7D reported by Watkins, Li [11]. Yet, attempts to find significant colocations and to associated those to existing literature of relevant traits including also other plants in the *Poaceae* family (but excluding studies on esterase involvement in pollen formation and disease resistance) did not yet yield convincing outcomes and associations, and therefore, to confirm the genes, future research can focus on sequencing the selected genes and the activities of the expressed protein.

**Table 2:** Genes putatively involved in the genetic determination of esterase and lipase.

Trait	Chromosome	Left most SNP	Gene ID	Gene annotation	-log_10_(*p*) (2015)	-log_10_(*p*) (2016)
esterase	2A	Tdurum_contig53038_684	TraesCS2A01G551500	alpha/beta-Hydrolases superfamily protein	-	7.9
esterase	2B	RAC875_c25271_138	TraesCS2B01G571800	Triacylglycerol lipase 2, putative	-	7.5
esterase	2B	RAC875_rep_c112916_263	TraesCS2B01G573900	GDSL esterase/lipase	-	8.0
esterase	2B	BobWhite_c33464_133	TraesCS2B01G579900	Lecithin-cholesterol acyltransferase-like 1	-	8.2
esterase	2D	Ku_c19185_1569	TraesCS2D01G360100	GDSL esterase/lipase	-	7.1
esterase	2D	Ku_c19185_1569	TraesCS2D01G362500	Lipase	-	7.1
esterase	2D	BobWhite_c36548_98	TraesCS2D01G542700	Triacylglycerol lipase 2, putative	-	7.7
esterase	2D	D_GBUVHFX01BGETX_77	TraesCS2D01G550200	Lecithin-cholesterol acyltransferase-like 1	-	8.3
lipase	3B	BS00093891_51	TraesCS3B01G445600	GDSL esterase/lipase	5.7	1.8
lipase	3B	BS00048754_51	TraesCS3B01G445700	GDSL esterase/lipase	4.8	1.1
lipase	4A	RFL_Contig3841_2409	TraesCS4A01G445000	GDSL esterase/lipase	-	6.1
lipase	6D	RFL_Contig4441_505	TraesCS6D01G108200	Sn1-specific diacylglycerol lipase alpha	2.4	5.0

The table shows the trait, chromosome, the first marker of the haplotype block, gene ID, annotation, and the significance value (p) for 2015 and 2016 when <0.01.

In conclusion, a large diversity for esterase and lipase activity was observed in the 300 wheat cultivars by an up to 2.5-fold difference, indicating genetic variability for these traits. Yet the environment also had large influences on the enzyme activities. The cultivars ‘Julius’ and ‘Bueno’ demonstrated low activities in both years, and therefore are potentially more suitable for stable wholegrain use. Finally, candidate genes underlying the QTL for esterase and lipase activities provide insight into the genetic architecture of these traits. Our study is the first to map QTL for lipase in wheat grain. The findings built an important foundation to locate the genetic source of the lipid hydrolytic enzymes. It provides opportunities for marker-assisted breeding of low-esterase/lipase wheat cultivars that are suitable for stable wholegrain flour with extended shelf life.

## Supporting information

S1 Table Esterase and lipase activities of 300 wheat cultivars harvested in 2015 and 2016. Non-starch lipid content of 300 wheat cultivars harvested in 2016.
(PDF)

S2 Table Significant SNP markers that pass the Bonferroni corrected LOD limit. The table shows the trait, chromosome, the first and second marker positions, the marker triplet, LOD and the variance explained (adjusted R^2^).
(PDF)
