# Supplementary material for: Genome-wide association study of lipase and esterase in wholegrain wheat flour (Triticum aestivum L.)
Source: PLoS One. 2023 Mar 9;18(3):e0282510. doi: 10.1371/journal.pone.0282510 (PMC9997868; doi:10.1371/journal.pone.0282510)
Supplement: S1 Table — Non-starch lipid content of 300 wheat cultivars harvested in 2016. (PDF) [file pone.0282510.s001.pdf]

Supporting information

Genetic determination of lipase and esterase activities in wheat (*Triticum aestivum L.*)

Short title: GWAS of lipase and esterase in wholegrain wheat flour

Chun Yue Wei [1], Steven Yates [2], Dan Zhu [1], Andreas Hund [3], Bruno Studer [2], Laura Nyström [1*]

[1] Laboratory of Food Biochemistry, Institute of Food, Nutrition and Health, ETH Zurich, Zurich, Switzerland

[2] Molecular Plant Breeding, Institute of Agricultural Sciences, ETH Zurich, Zurich, Switzerland

[3] Crop Science, Institute of Agricultural Sciences, ETH Zurich, Zurich, Switzerland

* Corresponding author

Email: laura.nystroem@hest.ethz.ch (LN)

S1 Table. Esterase and lipase activities of 300 wheat cultivars harvested in 2015 and 2016. Non-starch lipid content of 300 wheat cultivars harvested in 2016.

	Esterase (U/kg)		Lipase (U/kg)		Lipid (%)
Genotype	2015	2016	2015	2016	2016.00
TAMARO	983.1 ± 25.2	805.9 ± 31.2	22.7 ± 0.9	17.0 ± 0.4	1.44 ± 0.05
LUDWIG	891.8 ± 44.2	751.1 ± 23.1	21.7 ± 1.2	15.8 ± 0.5	1.52 ± 0.07
LEVIS	968.8 ± 67.4	782.5 ± 37.0	20.8 ± 1.4	14.6 ± 0.5	1.38 ± 0.14
ARINA	833.4 ± 35.1	748.4 ± 33.4	21.8 ± 0.6	15.4 ± 0.4	1.52 ± 0.04
APACHE	1011.1 ± 48.6	871.7 ± 32.0	22.1 ± 0.9	17.3 ± 0.4	1.41 ± 0.03
FORNO	1137.7 ± 38.7	984.8 ± 20.7	25.2 ± 0.8	20.4 ± 0.8	1.38 ± 0.11
CAMBRENA	883.1 ± 19.5	848.3 ± 29.6	20.9 ± 0.9	18.6 ± 0.4	1.35 ± 0.02
CAPHORN	1115.4 ± 59.9	1019.1 ± 54.9	23.5 ± 1.4	20.7 ± 0.6	1.34 ± 0.02
MULAN	1018.5 ± 55.8	953.0 ± 16.7	22.5 ± 0.8	19.9 ± 0.5	1.53 ± 0.01
GALAXIE	821.1 ± 53.6	808.9 ± 18.2	20.3 ± 0.6	18.0 ± 0.9	1.50 ± 0.01
TITLIS	911.4 ± 56.9	895.5 ± 30.7	19.9 ± 1.3	17.2 ± 0.4	1.45 ± 0.01
CH COMBIN	944.9 ± 55.8	820.6 ± 26.5	23.6 ± 1.0	15.1 ± 0.7	1.36 ± 0.00
CH CAMEDO	1087.3 ± 58.2	880.1 ± 30.4	16.9 ± 0.5	15.3 ± 0.9	1.33 ± 0.16
ZINAL	760.5 ± 22.0	812.7 ± 22.3	21.4 ± 1.0	18.8 ± 0.5	1.50 ± 0.04
FOREL	988.9 ± 35.9	639.4 ± 26.6	18.1 ± 0.8	16.1 ± 0.7	1.33 ± 0.04
SURETTA	991.4 ± 35.5	763.1 ± 24.3	18.0 ± 0.7	19.3 ± 0.3	1.30 ± 0.03
RUNAL	1034.1 ± 36.1	729.4 ± 40.1	16.7 ± 1.2	20.0 ± 0.5	1.48 ± 0.01
ACTROS	1151.9 ± 39.7	1038.7 ± 15.3	27.7 ± 0.8	21.1 ± 0.5	1.44 ± 0.03
AKTEUR	945.6 ± 84.7	737.7 ± 37.7	19.2 ± 0.4	17.1 ± 0.6	1.42 ± 0.02
BATIS	902.6 ± 39.9	724.2 ± 37.7	18.7 ± 0.6	18.5 ± 0.7	1.31 ± 0.01
BISCAY	1218.4 ± 86.3	1000.7 ± 26.8	19.1 ± 0.8	22.0 ± 0.9	1.30 ± 0.01
BOOMER	1046.4 ± 56.4	942.0 ± 55.9	20.8 ± 1.0	22.1 ± 1.5	1.43 ± 0.01
BORNEO	1131.4 ± 58.4	932.9 ± 65.8	20.7 ± 0.6	21.7 ± 1.3	1.62 ± 0.01
BRILLIANT	782.6 ± 43.3	771.1 ± 16.2	20.8 ± 1.0	17.1 ± 0.6	1.46 ± 0.02
BUSSARD	885.0 ± 33.6	723.9 ± 21.9	22.6 ± 0.7	16.5 ± 0.6	1.48 ± 0.00
BUTEO	1058.0 ± 57.1	911.5 ± 43.9	19.7 ± 0.8	25.0 ± 1.3	1.43 ± 0.02
CAPNOR	912.6 ± 32.8	921.7 ± 42.7	23.0 ± 0.6	18.7 ± 0.5	1.32 ± 0.03
CARENIUS	872.2 ± 48.7	818.4 ± 49.5	17.2 ± 1.1	22.7 ± 2.5	1.28 ± 0.03
CERTO	1030.6 ± 39.9	909.5 ± 42.3	18.7 ± 1.0	19.5 ± 1.3	1.46 ± 0.02
CENTRUM	1025.7 ± 41.9	810.3 ± 42.1	25.0 ± 1.2	22.1 ± 0.6	1.50 ± 0.01
COMPLIMENT	1026.8 ± 15.4	848.8 ± 29.5	25.6 ± 0.6	22.8 ± 1.3	1.57 ± 0.01
CETUS	812.0 ± 16.2	824.4 ± 28.4	24.5 ± 0.7	19.1 ± 0.3	1.54 ± 0.02
CUBUS	1041.3 ± 40.3	790.9 ± 50.9	24.5 ± 0.6	24.3 ± 2.4	1.37 ± 0.05
DISCUS	1013.3 ± 51.4	820.1 ± 29.4	21.6 ± 1.0	21.6 ± 0.8	1.61 ± 0.03
DEKAN	955.7 ± 36.4	757.7 ± 42.1	21.3 ± 0.7	21.8 ± 0.7	1.41 ± 0.00
DRIFTER	1175.7 ± 44.7	948.0 ± 39.4	27.0 ± 0.9	27.6 ± 1.1	1.50 ± 0.01
ENORM	821.6 ± 16.9	814.3 ± 31.7	19.7 ± 0.9	17.4 ± 0.8	1.31 ± 0.01
ELLVIS	950.2 ± 21.5	715.4 ± 45.6	21.3 ± 0.6	21.1 ± 0.3	1.35 ± 0.01
FEDOR	1040.3 ± 36.4	914.0 ± 23.6	26.3 ± 1.3	20.9 ± 0.7	1.67 ± 0.04
ESKET	1036.6 ± 32.1	865.0 ± 43.3	24.6 ± 0.7	24.1 ± 0.7	1.53 ± 0.04
FLAIR	892.0 ± 16.7	723.4 ± 40.3	22.9 ± 0.7	24.0 ± 1.0	1.15 ± 0.02
FORMAT	844.5 ± 43.1	868.0 ± 23.2	21.0 ± 0.8	19.1 ± 0.9	1.44 ± 0.02
GREIF	984.7 ± 29.5	710.5 ± 31.4	23.8 ± 0.5	21.8 ± 0.8	1.18 ± 0.02
HALDOR	1001.3 ± 57.4	855.1 ± 39.9	22.5 ± 2.0	24.1 ± 1.0	1.52 ± 0.01
HERMANN	1061.2 ± 42.5	926.1 ± 39.5	24.4 ± 0.9	24.1 ± 0.6	1.51 ± 0.02

HEROLDO	973.7 ± 49.1	842.3 ± 33.5	22.1 ± 1.6	20.8 ± 0.8	1.36 ± 0.02
IMPRESSION	1048.6 ± 24.6	803.6 ± 25.6	23.4 ± 0.9	20.0 ± 0.8	1.43 ± 0.02
INSPIRATION	1004.7 ± 29.5	910.3 ± 63.8	23.7 ± 1.5	24.9 ± 0.8	1.36 ± 0.01
JAFET	903.7 ± 22.7	811.8 ± 30.6	22.6 ± 0.8	19.8 ± 0.6	1.33 ± 0.01
JENGA	1109.7 ± 50.5	916.5 ± 28.1	23.0 ± 0.9	22.8 ± 0.6	1.60 ± 0.01
KOCH	992.2 ± 49.3	844.7 ± 32.9	23.5 ± 1.3	24.5 ± 1.0	1.58 ± 0.10
LEIFFER	1014.4 ± 96.5	928.6 ± 18.2	21.9 ± 1.0	23.8 ± 0.7	1.48 ± 0.02
LUCIUS	868.6 ± 67.8	746.7 ± 41.1	22.4 ± 1.1	24.4 ± 0.6	1.17 ± 0.02
MAGISTER	849.9 ± 31.0	823.9 ± 25.3	19.5 ± 0.8	19.1 ± 1.0	1.43 ± 0.00
MANAGER	748.5 ± 24.6	729.9 ± 19.6	21.4 ± 1.0	19.6 ± 1.0	1.22 ± 0.03
MANDUB	1129.0 ± 42.4	868.1 ± 58.5	21.3 ± 1.1	21.1 ± 0.8	1.56 ± 0.03
METEOR	927.1 ± 65.2	890.8 ± 68.7	19.8 ± 1.0	21.3 ± 0.6	1.50 ± 0.12
MEUNIER	883.2 ± 20.1	762.8 ± 20.1	22.2 ± 1.3	16.6 ± 0.5	1.33 ± 0.01
MILVUS	844.6 ± 33.4	747.7 ± 33.1	18.3 ± 1.4	20.5 ± 0.9	1.23 ± 0.02
MIRAGE	1039.6 ± 41.4	837.7 ± 48.6	22.4 ± 0.9	23.5 ± 1.6	1.39 ± 0.01
MONOPOL	990.0 ± 29.8	734.7 ± 49.6	22.0 ± 0.9	20.2 ± 0.8	1.50 ± 0.01
NATURASTAR	837.2 ± 44.0	693.6 ± 40.7	20.7 ± 0.6	14.6 ± 0.6	1.30 ± 0.01
OLIVIN	1024.8 ± 47.6	806.0 ± 44.1	22.2 ± 1.2	22.0 ± 0.9	1.52 ± 0.02
PAMIER	1037.6 ± 72.7	952.3 ± 32.8	18.8 ± 1.1	22.8 ± 0.7	1.31 ± 0.06
PAROLI	1040.3 ± 52.4	951.6 ± 34.7	22.7 ± 2.1	26.3 ± 0.5	1.37 ± 0.02
PETRUS	885.5 ± 31.4	770.5 ± 21.3	22.2 ± 0.9	16.6 ± 0.5	1.60 ± 0.04
POTENZIAL	1039.1 ± 49.3	927.0 ± 39.1	19.9 ± 0.7	19.9 ± 0.6	1.64 ± 0.00
PRIVILEG	948.3 ± 27.2	868.1 ± 50.9	20.0 ± 1.4	22.0 ± 0.7	1.33 ± 0.03
PROFILUS	868.8 ± 40.6	798.6 ± 37.9	24.0 ± 0.8	16.6 ± 0.7	1.37 ± 0.02
QUEBON	976.5 ± 64.3	825.9 ± 56.4	20.4 ± 1.0	19.2 ± 0.6	1.26 ± 0.01
RETRO	1014.1 ± 36.9	944.9 ± 17.6	21.7 ± 0.7	16.7 ± 0.4	1.53 ± 0.01
ROMANUS	1065.7 ± 23.4	1029.3 ± 18.3	21.0 ± 0.7	27.8 ± 0.8	1.46 ± 0.02
SCHAMANE	1214.6 ± 43.3	962.6 ± 40.8	20.6 ± 0.9	21.7 ± 0.4	1.78 ± 0.03
SKAGEN	970.9 ± 48.8	874.6 ± 36.5	18.0 ± 0.8	19.7 ± 1.0	1.62 ± 0.02
SKALMEJE	780.9 ± 38.5	795.0 ± 25.5	18.6 ± 0.5	14.4 ± 0.5	1.27 ± 0.02
SKATER	1076.5 ± 42.3	863.5 ± 26.4	19.1 ± 0.9	22.9 ± 0.9	1.40 ± 0.03
SOBI	984.3 ± 26.0	763.3 ± 45.4	19.4 ± 1.2	21.5 ± 0.7	1.23 ± 0.01
SOKRATES	930.6 ± 29.7	725.8 ± 50.8	16.8 ± 0.2	19.5 ± 0.6	1.28 ± 0.04
SOLITÄR	944.5 ± 27.3	780.0 ± 56.5	18.5 ± 0.8	20.4 ± 1.1	1.30 ± 0.01
SOPHYTRA	895.8 ± 22.6	773.9 ± 31.9	17.4 ± 1.2	18.1 ± 0.8	1.31 ± 0.02
STRIKER	1018.2 ± 43.3	888.3 ± 39.2	19.9 ± 0.7	22.8 ± 0.7	1.46 ± 0.02
SW TOPPER	1170.3 ± 33.9	968.0 ± 49.4	19.8 ± 0.4	21.0 ± 0.9	1.62 ± 0.03
TABASCO	1121.1 ± 75.4	901.1 ± 23.1	20.0 ± 1.0	23.6 ± 0.4	1.41 ± 0.00
TAMBOR	1082.1 ± 50.3	895.7 ± 63.6	18.5 ± 1.0	24.1 ± 0.8	1.60 ± 0.02
TORAS	1085.9 ± 33.8	843.4 ± 43.0	16.4 ± 0.8	23.5 ± 1.2	1.67 ± 0.02
TORONTO	1110.9 ± 43.9	895.3 ± 51.1	19.8 ± 0.6	25.6 ± 0.9	1.46 ± 0.00
TRANSIT	1097.4 ± 41.6	846.9 ± 62.7	21.8 ± 0.9	24.7 ± 0.9	1.35 ± 0.01
TUAREG	1030.4 ± 24.9	845.7 ± 62.0	17.8 ± 1.0	21.2 ± 1.3	1.56 ± 0.01
TUKAN	1023.5 ± 28.2	893.7 ± 50.2	18.4 ± 1.3	27.4 ± 0.4	1.44 ± 0.01
TULSA	1004.9 ± 44.9	892.0 ± 35.2	19.3 ± 0.9	23.4 ± 1.0	1.52 ± 0.00
WINNETOU	987.4 ± 35.1	806.9 ± 66.8	17.4 ± 1.0	23.3 ± 0.8	1.46 ± 0.02
ZENTOS	1062.0 ± 30.3	825.9 ± 30.0	18.6 ± 0.6	27.3 ± 0.7	1.65 ± 0.02
ZOBEL	1038.9 ± 27.7	785.5 ± 52.7	19.2 ± 1.3	22.8 ± 0.6	1.52 ± 0.02
CLIFF	1129.7 ± 50.6	873.7 ± 47.9	19.1 ± 1.5	28.7 ± 0.9	1.45 ± 0.02
HISTORY	1062.3 ± 22.6	805.9 ± 37.5	20.1 ± 0.7	21.8 ± 0.5	1.40 ± 0.05

LINDOS	1072.6 ± 30.6	794.3 ± 47.2	18.2 ± 0.8	27.2 ± 0.5	1.26 ± 0.02
JULIUS	765.9 ± 45.0	774.4 ± 27.2	16.2 ± 0.8	12.6 ± 0.5	1.26 ± 0.03
TIGER	914.7 ± 51.5	725.3 ± 42.4	20.0 ± 0.8	20.4 ± 0.6	1.44 ± 0.01
EXOTIC	920.2 ± 41.6	810.6 ± 29.4	23.6 ± 0.9	16.3 ± 0.7	1.35 ± 0.01
LOCH 3754					
ADLON	1021.6 ± 43.8	1034.3 ± 32.1	20.8 ± 1.1	30.2 ± 1.2	1.49 ± 0.02
ACIENDA	1033.8 ± 49.8	942.5 ± 28.6	21.3 ± 0.9	28.8 ± 1.6	1.51 ± 0.01
AGUILA	1025.0 ± 51.7	841.0 ± 28.0	19.4 ± 1.3	22.7 ± 0.8	1.33 ± 0.02
ALLISTER	1019.3 ± 31.4	822.0 ± 21.7	19.7 ± 0.9	21.1 ± 0.9	1.45 ± 0.03
ARACK	1085.0 ± 25.0	778.4 ± 34.9	23.4 ± 1.0	21.3 ± 0.7	1.45 ± 0.01
AROBASE	1020.1 ± 36.9	922.9 ± 48.9	21.9 ± 1.3	23.8 ± 1.8	1.33 ± 0.01
ATTLASS	726.8 ± 44.8	846.7 ± 34.4	20.1 ± 0.3	24.4 ± 0.8	1.66 ± 0.00
AUTAN	678.6 ± 51.8	897.6 ± 28.6	19.2 ± 0.5	22.6 ± 1.4	1.47 ± 0.01
AVANTAGE	684.8 ± 41.9	897.1 ± 33.1	19.0 ± 0.4	26.3 ± 1.1	1.44 ± 0.01
AZIMUT	892.5 ± 37.3	979.8 ± 37.7	22.7 ± 0.8	31.0 ± 0.8	1.45 ± 0.02
AZZURO	761.6 ± 25.5	918.3 ± 32.6	20.8 ± 1.1	30.3 ± 0.8	1.42 ± 0.01
BAGATELLE 007	802.2 ± 38.9	805.2 ± 53.9	20.4 ± 0.4	22.7 ± 2.0	1.59 ± 0.03
BALANCE	959.2 ± 31.7	1104.4 ± 61.8	21.6 ± 0.6	31.4 ± 0.8	1.49 ± 0.02
BALTIMOR	716.4 ± 34.2	926.3 ± 30.8	19.3 ± 0.7	24.9 ± 1.2	1.66 ± 0.01
BASTIDE	856.0 ± 45.7	880.8 ± 33.5	18.7 ± 1.1	32.7 ± 2.0	1.37 ± 0.01
BRANDO	1036.9 ± 64.7	969.2 ± 29.8	24.9 ± 2.2	19.1 ± 1.3	1.38 ± 0.00
CALISTO	873.0 ± 25.4	768.7 ± 20.6	21.3 ± 0.8	13.9 ± 0.2	1.38 ± 0.01
CAMPERO	904.1 ± 27.5	930.7 ± 26.4	18.5 ± 0.3	28.6 ± 1.2	1.32 ± 0.02
CARIBOU	1034.1 ± 37.5	923.2 ± 35.1	20.8 ± 0.9	26.6 ± 0.7	1.55 ± 0.03
CATALAN	853.9 ± 35.6	739.4 ± 66.4	18.7 ± 0.8	14.2 ± 0.9	1.30 ± 0.03
CEZANNE	1035.3 ± 36.0	902.7 ± 53.0	21.6 ± 0.7	30.4 ± 1.0	1.40 ± 0.01
CHARGER	972.9 ± 54.9	900.2 ± 46.8	19.3 ± 0.9	23.5 ± 1.0	1.44 ± 0.01
CRAKLIN	904.2 ± 38.0	802.3 ± 28.7	22.9 ± 0.5	13.9 ± 0.9	1.44 ± 0.01
DINOSOR	999.2 ± 44.0	915.0 ± 49.8	19.4 ± 0.5	26.9 ± 0.7	1.41 ± 0.01
EPIDOC	845.8 ± 33.5	768.3 ± 29.4	18.7 ± 1.0	12.7 ± 0.5	1.38 ± 0.04
EQUINOX	1022.9 ± 37.5	933.1 ± 37.7	20.7 ± 0.7	23.6 ± 0.8	1.29 ± 0.03
FOLIO	921.9 ± 23.2	841.2 ± 36.4	19.6 ± 0.7	22.1 ± 1.1	1.37 ± 0.01
FORBAN	990.1 ± 35.7	778.3 ± 21.4	19.9 ± 0.9	20.7 ± 0.4	1.23 ± 0.02
GARCIA	850.6 ± 41.0	875.2 ± 30.3	19.8 ± 0.7	15.9 ± 0.5	1.46 ± 0.02
GRISBY	1060.1 ± 35.4	1069.5 ± 30.2	19.0 ± 1.5	29.7 ± 0.7	1.38 ± 0.01
HAMAC	946.4 ± 54.9	770.1 ± 24.9	20.4 ± 0.8	15.9 ± 1.0	1.59 ± 0.01
HAUSSMANN	972.0 ± 41.9	830.1 ± 36.2	20.2 ± 0.7	19.7 ± 1.0	1.29 ± 0.01
INCISIF	1057.2 ± 29.8	938.8 ± 40.8	18.2 ± 0.5	21.5 ± 0.7	1.41 ± 0.01
INOUI	1044.7 ± 50.8	950.5 ± 43.6	17.9 ± 0.8	21.8 ± 0.9	1.53 ± 0.02
RICHEPAIN	976.3 ± 54.1	783.5 ± 38.7	17.2 ± 0.9	19.6 ± 0.8	1.26 ± 0.02
INSTINCT	1047.1 ± 49.6	986.1 ± 32.0	17.5 ± 0.5	22.7 ± 0.6	1.38 ± 0.03
INTACT	1222.8 ± 44.2	948.9 ± 25.0	20.6 ± 1.3	23.2 ± 0.5	1.55 ± 0.02
INTENSE	914.9 ± 57.7	799.9 ± 27.7	18.5 ± 0.8	17.0 ± 0.4	1.45 ± 0.01
ISENGRAIN	1030.7 ± 37.7	814.2 ± 26.9	20.5 ± 0.8	19.4 ± 0.7	1.33 ± 0.02
KLEBER	1035.8 ± 52.3	943.3 ± 54.3	22.7 ± 0.4	27.3 ± 0.8	1.30 ± 0.02
LONA	891.6 ± 26.1	838.7 ± 32.3	16.3 ± 0.9	14.9 ± 0.4	1.37 ± 0.01
MACRO	1041.8 ± 49.0	921.2 ± 32.6	19.0 ± 0.9	25.7 ± 0.8	1.32 ± 0.01
MARCHEVAL	888.4 ± 34.8	780.2 ± 44.1	16.9 ± 0.6	21.2 ± 0.7	1.27 ± 0.01
MAXYL	1006.5 ± 43.2	922.0 ± 44.0	18.6 ± 0.6	20.2 ± 0.9	1.35 ± 0.02
MENDEL	945.4 ± 53.4	938.6 ± 38.9	17.2 ± 0.9	23.2 ± 0.9	1.40 ± 0.01

MERCATO	787.9 ± 26.5	752.1 ± 26.0	21.7 ± 1.8	20.7 ± 0.6	1.46 ± 0.03
NIRVANA	884.1 ± 36.4	760.9 ± 39.2	15.6 ± 0.9	19.8 ± 0.8	1.21 ± 0.02
OCTET	1003.2 ± 47.4	878.4 ± 42.5	20.2 ± 0.5	23.1 ± 0.7	1.41 ± 0.01
ORDEAL	1020.2 ± 25.5	983.2 ± 28.4	20.0 ± 0.9	23.2 ± 0.5	1.45 ± 0.03
ORNICAR	814.6 ± 28.3	769.4 ± 32.4	20.2 ± 0.8	23.5 ± 0.8	1.55 ± 0.02
PALADAIN	846.1 ± 40.6	846.5 ± 23.7	18.0 ± 1.0	18.6 ± 0.9	1.36 ± 0.11
PARADOR	951.7 ± 45.3	877.5 ± 37.1	17.8 ± 0.7	23.3 ± 2.7	1.30 ± 0.03
PR 22 R 28	851.7 ± 75.9	823.0 ± 54.0	16.9 ± 0.8	23.7 ± 0.7	1.17 ± 0.02
PR22R20	1063.7 ± 63.6	912.2 ± 63.3	20.6 ± 0.8	22.2 ± 0.8	1.22 ± 0.01
PULSAR	845.5 ± 33.9	860.8 ± 40.5	16.4 ± 0.8	19.4 ± 0.7	1.40 ± 0.00
PYTAGOR	880.1 ± 48.0	822.0 ± 27.0	17.8 ± 0.6	19.5 ± 1.0	1.29 ± 0.01
QUATUOR	966.7 ± 50.7	958.3 ± 28.5	19.9 ± 0.9	24.3 ± 1.0	1.32 ± 0.01
RAISON	1082.4 ± 23.9	1064.7 ± 25.2	21.9 ± 0.7	24.8 ± 0.5	1.60 ± 0.01
ROYSSAC	974.5 ± 48.4	906.9 ± 25.6	19.9 ± 1.2	23.6 ± 0.7	1.37 ± 0.01
SAMURAI	968.1 ± 44.4	914.6 ± 39.5	17.5 ± 0.6	20.8 ± 1.0	1.30 ± 0.02
SANKARA	857.3 ± 44.8	867.2 ± 47.6	17.0 ± 1.0	21.6 ± 0.5	1.40 ± 0.01
SEMAFOR	1048.7 ± 88.3	920.2 ± 41.4	17.8 ± 1.2	21.6 ± 1.4	1.50 ± 0.02
SEYRAC	926.7 ± 34.3	885.4 ± 38.1	18.1 ± 1.0	23.4 ± 0.3	1.51 ± 0.01
SISLEY	948.5 ± 26.7	862.8 ± 17.2	19.2 ± 0.7	23.0 ± 1.1	1.37 ± 0.04
TOISONDOR	902.0 ± 51.2	783.3 ± 28.7	22.7 ± 1.2	19.9 ± 1.1	1.53 ± 0.01
TROCADERO	986.0 ± 32.7	814.7 ± 18.4	19.2 ± 1.1	14.9 ± 0.4	1.35 ± 0.02
VERSAILLES	1062.0 ± 43.1	1016.7 ± 36.5	19.2 ± 0.8	26.1 ± 0.9	1.46 ± 0.01
CCB INGÉNIO	914.0 ± 38.8	791.8 ± 31.9	15.4 ± 1.4	20.8 ± 0.7	1.57 ± 0.02
INÉDIT	842.0 ± 44.9	816.4 ± 28.8	19.3 ± 1.3	17.9 ± 0.6	1.24 ± 0.01
CCB PRÉFÉRENCE	895.0 ± 42.9	801.1 ± 64.9	14.9 ± 0.8	24.7 ± 0.9	1.34 ± 0.01
INTÉRÊT	751.2 ± 39.8	776.9 ± 44.2	18.3 ± 0.9	15.1 ± 0.7	1.21 ± 0.01
EXPERT	913.8 ± 26.7	793.6 ± 61.2	13.6 ± 0.9	22.4 ± 0.8	1.42 ± 0.00
BUENO	782.7 ± 28.5	638.0 ± 39.2	13.6 ± 1.3	18.9 ± 0.5	1.30 ± 0.05
H05581A	798.5 ± 32.1	725.5 ± 22.7	17.8 ± 0.5	13.3 ± 0.5	1.27 ± 0.02
H04438	988.7 ± 37.5	823.7 ± 58.0	15.2 ± 0.9	20.0 ± 1.0	1.31 ± 0.02
H03309	829.9 ± 28.6	722.7 ± 17.8	20.3 ± 1.0	16.9 ± 0.6	1.51 ± 0.01
INNOV	877.8 ± 97.3	865.9 ± 45.2	16.1 ± 0.6	23.6 ± 0.7	1.53 ± 0.00
ORATORIO	988.1 ± 40.2	976.2 ± 56.6	16.3 ± 0.6	26.3 ± 1.0	1.54 ± 0.02
RECITAL	1057.0 ± 70.9	845.7 ± 32.9	25.7 ± 1.8	18.7 ± 0.5	1.46 ± 0.03
PR22R58	901.0 ± 37.8	838.2 ± 56.0	13.9 ± 0.9	19.8 ± 1.1	1.42 ± 0.02
ASTRADO	856.8 ± 28.1	792.7 ± 29.3	16.0 ± 1.2	19.9 ± 0.6	1.40 ± 0.03
BERMUDE	913.1 ± 37.6	1035.9 ± 38.7	16.1 ± 0.8	22.8 ± 0.9	1.34 ± 0.02
EUCLIDE	965.4 ± 31.2	977.8 ± 41.4	16.0 ± 1.0	22.7 ± 0.4	1.43 ± 0.03
ALDRIC	880.8 ± 45.0	927.9 ± 43.6	17.0 ± 1.1	28.4 ± 1.2	1.33 ± 0.01
ALTIGO	1020.4 ± 35.1	887.4 ± 22.8	25.9 ± 0.9	16.7 ± 0.6	1.38 ± 0.01
IRIDIUM	796.3 ± 83.2	849.0 ± 31.9	13.9 ± 1.1	22.2 ± 0.9	1.33 ± 0.00
AUDI	1009.8 ± 65.9	962.6 ± 70.5	16.0 ± 0.8	24.7 ± 0.6	1.61 ± 0.03
BILL	901.2 ± 41.0	860.8 ± 42.2	15.9 ± 1.3	23.2 ± 1.2	1.33 ± 0.01
CASSIOPEIA	1041.3 ± 33.3	1035.6 ± 52.0	20.7 ± 1.1	30.3 ± 0.9	1.54 ± 0.02
CONTUR	869.5 ± 21.3	902.7 ± 26.2	17.9 ± 0.6	28.2 ± 0.8	1.60 ± 0.01
FASTNET	883.1 ± 37.4	820.7 ± 35.7	15.2 ± 0.2	22.7 ± 0.6	1.30 ± 0.01
HEREFORD	888.9 ± 43.0	846.2 ± 59.0	16.4 ± 0.9	22.5 ± 0.5	1.44 ± 0.01
PORTLAND	922.6 ± 44.5	878.9 ± 42.1	15.6 ± 1.0	22.2 ± 1.2	1.29 ± 0.02
SENAT	934.6 ± 24.0	846.3 ± 24.7	23.4 ± 1.3	16.4 ± 0.6	1.32 ± 0.01

SMUGGLER	984.9 ± 55.9	1022.0 ± 35.5	17.2 ± 1.0	27.8 ± 0.9	1.50 ± 0.03
TRINTELLA	984.7 ± 34.4	940.4 ± 43.1	16.2 ± 1.1	25.8 ± 0.7	1.56 ± 0.01
ZANATAN	838.4 ± 29.6	829.6 ± 19.3	15.2 ± 0.8	20.9 ± 0.6	1.32 ± 0.00
ACHAT	1029.7 ± 31.3	901.5 ± 35.8	18.8 ± 1.4	21.1 ± 0.7	1.59 ± 0.01
CAPO	929.9 ± 26.0	724.3 ± 19.3	21.6 ± 1.5	15.6 ± 0.7	1.35 ± 0.01
ELEMENT	849.6 ± 41.9	834.6 ± 29.2	22.1 ± 0.5	20.0 ± 0.6	1.59 ± 0.04
ERIWAN	854.4 ± 46.9	1062.1 ± 60.1	20.7 ± 0.7	24.8 ± 0.8	1.86 ± 0.05
EUROJET	824.5 ± 49.4	913.4 ± 31.5	20.2 ± 1.0	23.5 ± 1.0	1.78 ± 0.02
FRIDOLIN	700.3 ± 50.7	675.8 ± 31.4	17.9 ± 0.6	15.6 ± 0.3	1.46 ± 0.03
GLOBUS	708.3 ± 33.9	738.7 ± 36.0	18.4 ± 0.8	20.0 ± 0.7	1.18 ± 0.01
RAINER	996.9 ± 37.4	867.8 ± 19.8	22.1 ± 1.0	16.6 ± 1.2	1.59 ± 0.01
VITUS	946.6 ± 34.6	823.6 ± 25.7	23.5 ± 1.0	17.0 ± 0.5	1.45 ± 0.01
XENOS	798.7 ± 48.4	777.0 ± 32.3	19.8 ± 0.7	19.9 ± 0.6	1.43 ± 0.13
BANQUET	1023.6 ± 32.6	799.6 ± 27.8	23.8 ± 0.6	17.3 ± 0.6	1.49 ± 0.04
ESTICA	857.2 ± 33.1	851.7 ± 25.7	20.4 ± 0.5	22.9 ± 0.8	1.34 ± 0.02
SEMPER	904.3 ± 23.0	782.3 ± 64.5	21.4 ± 0.9	19.3 ± 0.8	1.47 ± 0.01
BATUTA	720.3 ± 33.6	735.4 ± 38.9	18.2 ± 0.4	17.0 ± 1.0	1.44 ± 0.02
BOGATKA	731.0 ± 29.1	731.3 ± 38.7	18.1 ± 0.6	18.9 ± 0.5	1.49 ± 0.02
FIGURA	706.6 ± 52.1	796.7 ± 31.4	15.1 ± 0.6	20.5 ± 1.6	1.43 ± 0.02
FINEZJA	730.2 ± 42.8	768.3 ± 41.3	16.0 ± 0.7	20.8 ± 1.2	1.44 ± 0.01
FREGATA	702.2 ± 21.8	825.1 ± 49.6	14.0 ± 0.7	20.2 ± 0.8	1.60 ± 0.04
IZYDA	976.5 ± 35.9	803.0 ± 33.7	21.2 ± 0.8	13.8 ± 0.4	1.53 ± 0.02
KOBIERA	802.5 ± 31.0	857.0 ± 31.7	16.5 ± 0.7	21.6 ± 0.4	1.71 ± 0.03
KOBRA PLUS	741.6 ± 27.5	860.9 ± 39.1	16.3 ± 0.3	22.1 ± 0.5	1.76 ± 0.00
KORWETA	744.1 ± 26.1	904.5 ± 51.0	15.9 ± 0.7	23.9 ± 0.5	1.43 ± 0.02
LEGENDA	830.5 ± 43.5	860.3 ± 50.8	17.1 ± 0.9	20.0 ± 0.6	1.69 ± 0.02
MARKIZA	851.2 ± 36.5	970.7 ± 33.4	16.8 ± 0.4	25.9 ± 0.4	1.27 ± 0.03
MEWA	888.3 ± 25.0	714.0 ± 40.6	22.7 ± 0.5	15.5 ± 0.7	1.63 ± 0.02
NADOBNA	983.4 ± 34.3	802.0 ± 62.6	23.1 ± 0.8	16.7 ± 0.6	1.43 ± 0.00
NARIDANA	887.2 ± 21.2	963.6 ± 34.5	17.2 ± 1.0	25.6 ± 0.9	1.59 ± 0.05
NATEJA	852.1 ± 41.4	791.5 ± 38.5	17.4 ± 1.1	26.3 ± 0.6	1.51 ± 0.02
NUTKA	880.5 ± 42.7	807.9 ± 38.5	16.8 ± 1.2	24.8 ± 0.6	1.32 ± 0.00
OSTKA STRZELECKA	1005.7 ± 15.9	833.3 ± 53.4	24.1 ± 0.5	16.5 ± 0.7	1.57 ± 0.02
RYWALKA	815.7 ± 29.8	745.8 ± 28.7	16.9 ± 0.8	19.2 ± 0.9	1.55 ± 0.02
RUBENS	925.7 ± 15.9	786.2 ± 16.5	22.3 ± 0.6	16.5 ± 0.6	1.66 ± 0.03
SATYNA	839.7 ± 31.9	794.3 ± 44.7	16.2 ± 0.6	20.3 ± 0.9	1.13 ± 0.09
SMUGA	1011.2 ± 27.1	834.7 ± 31.0	22.5 ± 0.7	16.2 ± 0.5	1.49 ± 0.00
TONACJA	812.0 ± 29.6	848.0 ± 40.4	15.5 ± 0.6	20.8 ± 1.3	1.21 ± 0.03
WYDMA	706.5 ± 45.9	799.0 ± 32.7	20.4 ± 0.5	21.4 ± 1.3	1.33 ± 0.02
ZAWISZA	753.2 ± 29.4	775.0 ± 35.6	18.0 ± 0.4	22.0 ± 1.5	1.26 ± 0.03
ZYTA	731.0 ± 55.4	795.8 ± 35.6	17.0 ± 0.5	23.9 ± 0.7	1.32 ± 0.04
APERITIV	957.2 ± 33.5	775.3 ± 25.8	21.0 ± 1.6	16.1 ± 1.3	1.41 ± 0.01
KOSACK	1025.2 ± 40.5	808.4 ± 25.8	22.2 ± 1.2	17.3 ± 0.9	1.66 ± 0.01
LARS	878.1 ± 28.8	1018.3 ± 48.4	17.9 ± 0.6	23.3 ± 0.8	1.28 ± 0.01
MARSHAL	907.7 ± 61.2	1199.8 ± 63.7	18.7 ± 0.8	28.0 ± 1.3	1.48 ± 0.01
SW GNEJS	951.5 ± 22.8	957.9 ± 26.8	21.2 ± 0.4	20.3 ± 0.9	1.54 ± 0.00
SW HARNESK	824.3 ± 27.1	977.1 ± 51.3	19.6 ± 0.8	23.3 ± 1.6	1.49 ± 0.01
SW HARPUN	986.0 ± 27.9	1019.3 ± 19.8	26.2 ± 0.8	19.1 ± 0.4	1.72 ± 0.03
SW SKOTTE	814.2 ± 36.7	881.8 ± 61.0	18.5 ± 0.8	16.7 ± 0.8	1.46 ± 0.01

SW HURTIG	955.3 ± 37.7	1006.8 ± 32.7	19.5 ± 0.8	18.4 ± 0.7	1.62 ± 0.03
ALCHEMY	859.8 ± 32.3	969.0 ± 41.0	20.9 ± 0.9	26.1 ± 1.3	1.47 ± 0.01
AMBROSIA	705.0 ± 31.0	913.6 ± 24.5	16.9 ± 1.2	22.7 ± 1.3	1.44 ± 0.01
BROMPTON	881.5 ± 35.1	992.1 ± 27.4	19.3 ± 0.7	23.6 ± 1.0	1.44 ± 0.01
CONSORT	827.0 ± 32.5	904.6 ± 39.7	17.7 ± 1.1	20.4 ± 0.8	1.67 ± 0.02
CORDIALE	1002.6 ± 56.0	845.6 ± 29.4	25.0 ± 0.5	15.9 ± 0.7	1.46 ± 0.02
CPBT W130	776.5 ± 25.4	810.9 ± 52.0	18.6 ± 0.9	20.5 ± 0.6	1.47 ± 0.01
DEBEN	943.5 ± 42.9	1004.2 ± 48.6	18.8 ± 0.8	21.8 ± 1.0	1.63 ± 0.01
DUXFORD	935.9 ± 33.0	1098.3 ± 48.7	20.2 ± 1.3	24.5 ± 1.9	1.65 ± 0.02
EINSTEIN	739.2 ± 12.4	827.7 ± 42.3	17.1 ± 0.5	22.0 ± 0.4	1.39 ± 0.01
GATSBY	952.7 ± 48.1	955.2 ± 31.3	24.6 ± 1.4	22.1 ± 0.9	1.51 ± 0.02
GLADIATOR	790.8 ± 18.8	939.8 ± 50.2	17.7 ± 0.7	22.8 ± 0.8	1.35 ± 0.00
GLASGOW	1121.6 ± 29.4	881.2 ± 24.5	23.6 ± 1.6	15.8 ± 0.3	1.64 ± 0.01
GULLIVER	739.8 ± 22.4	995.5 ± 21.1	16.4 ± 0.9	26.0 ± 0.7	1.34 ± 0.01
HUMBER	730.5 ± 17.1	985.1 ± 34.8	15.1 ± 0.6	24.1 ± 0.7	1.37 ± 0.00
HYPERION	843.1 ± 39.1	930.0 ± 24.6	16.9 ± 0.7	21.2 ± 0.9	1.38 ± 0.01
ISTABRAQ	810.3 ± 20.1	847.1 ± 33.1	17.0 ± 0.7	19.5 ± 0.7	1.43 ± 0.01
MASCOT	808.7 ± 22.1	960.7 ± 40.4	16.5 ± 0.9	25.7 ± 0.7	1.47 ± 0.03
MUSKETEER	867.5 ± 33.8	1055.9 ± 47.4	19.9 ± 0.7	26.4 ± 0.9	1.37 ± 0.01
OAKLEY	949.8 ± 27.9	991.2 ± 15.3	20.6 ± 0.7	25.5 ± 1.0	1.69 ± 0.03
ROBIGUS	795.6 ± 27.8	877.1 ± 39.5	16.9 ± 1.2	22.5 ± 0.7	1.53 ± 0.01
SOISSONS	856.7 ± 21.3	896.2 ± 36.1	17.7 ± 0.7	22.6 ± 0.5	1.44 ± 0.02
VELOCITY	1137.5 ± 37.7	890.8 ± 41.1	21.4 ± 1.0	16.8 ± 0.6	1.57 ± 0.02
ZEBEDEE	795.2 ± 35.6	934.9 ± 35.6	17.1 ± 0.9	26.2 ± 0.9	1.46 ± 0.01
WELFORD	811.7 ± 39.3	920.8 ± 35.8	16.7 ± 1.1	22.4 ± 2.3	1.40 ± 0.00
SW TATAROS	860.2 ± 28.9	965.6 ± 24.1	15.8 ± 0.5	23.1 ± 1.0	1.66 ± 0.03
VIVANT	994.7 ± 24.1	1103.1 ± 40.0	20.0 ± 0.5	29.2 ± 1.4	1.41 ± 0.03
APOLLO	863.3 ± 28.3	872.2 ± 27.9	18.0 ± 0.6	21.8 ± 0.7	1.39 ± 0.01
ARES	690.5 ± 31.7	750.2 ± 15.7	17.9 ± 0.7	22.0 ± 0.4	1.51 ± 0.01
EXSEPT	750.6 ± 29.5	820.5 ± 23.1	18.2 ± 0.8	21.8 ± 0.6	1.43 ± 0.01
HANSEAT	785.9 ± 36.8	863.1 ± 48.2	17.8 ± 0.8	22.8 ± 0.6	1.39 ± 0.02
IBIS	740.3 ± 39.9	775.4 ± 26.9	15.7 ± 0.4	26.8 ± 0.5	1.52 ± 0.03
KANZLER	690.5 ± 31.4	810.0 ± 17.8	15.0 ± 0.6	23.4 ± 0.6	1.65 ± 0.00
MIKON	1148.1 ± 53.4	872.3 ± 26.2	20.3 ± 0.8	18.3 ± 0.5	1.85 ± 0.01
ORESTIS	880.2 ± 32.6	871.8 ± 23.9	19.6 ± 0.7	25.8 ± 0.8	1.85 ± 0.03
PRINZ	846.1 ± 38.4	924.7 ± 56.7	19.1 ± 0.6	28.1 ± 0.6	1.48 ± 0.01
RAMIRO	692.8 ± 22.7	679.7 ± 31.2	16.9 ± 0.9	20.6 ± 1.0	1.50 ± 0.01
RENAN	731.6 ± 39.8	746.6 ± 42.1	18.1 ± 0.4	20.9 ± 1.1	1.35 ± 0.04
SAVANNAH	877.3 ± 57.0	956.0 ± 43.6	18.4 ± 0.6	21.9 ± 0.6	1.65 ± 0.02
SLEJPNER	1120.7 ± 26.4	969.6 ± 26.0	18.2 ± 0.6	18.9 ± 0.8	1.45 ± 0.01
SPERBER	823.1 ± 35.5	982.2 ± 47.5	17.3 ± 0.7	27.4 ± 1.2	1.57 ± 0.02
TRAVIX	838.3 ± 46.6	967.9 ± 53.2	16.2 ± 0.8	21.1 ± 1.2	1.43 ± 0.03
VISCOUNT CPBT W 136	1197.5 ± 37.4	980.6 ± 25.0	20.3 ± 0.8	21.5 ± 0.8	1.49 ± 0.02
PHILIUS	834.9 ± 32.4	874.3 ± 51.7	16.3 ± 0.7	22.4 ± 1.1	1.38 ± 0.05
CAESAR	920.4 ± 30.8	922.8 ± 35.7	19.7 ± 0.7	22.4 ± 0.9	1.77 ± 0.01
HUSSAR	794.0 ± 45.2	958.3 ± 32.2	17.3 ± 0.7	21.5 ± 1.2	1.37 ± 0.00
URBAN	816.7 ± 33.2	944.5 ± 45.4	20.2 ± 1.0	25.6 ± 0.7	1.26 ± 0.03
MONSUN	1014.6 ± 57.2	855.1 ± 22.8	16.7 ± 1.0	16.2 ± 0.3	1.50 ± 0.03
TAIFUN	949.1 ± 35.5	850.9 ± 30.5	22.6 ± 1.0	14.8 ± 0.5	1.68 ± 0.01

MARIN	830.8 ± 28.2	914.6 ± 49.4	17.7 ± 0.8	21.3 ± 0.8	1.38 ± 0.04
KWS SCIROCCO LP 509.3.04	833.6 ± 24.6	832.6 ± 49.1	18.1 ± 0.3	20.2 ± 0.8	1.66 ± 0.01
KWS CHAMSIN LP 779.2.04	1101.6 ± 24.2	966.2 ± 28.7	17.5 ± 0.8	15.7 ± 0.8	1.62 ± 0.04
KWS AURUM LP 819.4.04	698.9 ± 42.8	848.8 ± 58.3	15.8 ± 0.5	20.2 ± 1.2	1.44 ± 0.03
VANEK	788.0 ± 42.3	816.9 ± 43.0	15.8 ± 0.5	20.1 ± 0.9	1.39 ± 0.01
TRAPPE	907.6 ± 34.8	1026.2 ± 44.2	17.4 ± 0.9	26.3 ± 1.1	1.60 ± 0.04
BRYZA	1146.6 ± 48.5	933.4 ± 37.3	19.2 ± 1.0	17.7 ± 0.6	1.62 ± 0.03
H05606	780.8 ± 28.2	821.7 ± 42.6	16.6 ± 0.6	23.2 ± 1.9	1.33 ± 0.02
P05312	783.0 ± 33.4	845.4 ± 20.4	16.6 ± 0.7	21.8 ± 2.2	1.36 ± 0.01
