# Supplementary material for: Genome-wide association study of lipase and esterase in wholegrain wheat flour (Triticum aestivum L.)
Source: PLoS One. 2023 Mar 9;18(3):e0282510. doi: 10.1371/journal.pone.0282510 (PMC9997868; doi:10.1371/journal.pone.0282510)
Supplement: S2 Table — The table shows the trait, chromosome, the first and second marker positions, the marker triplet, LOD and the variance explained (adjusted R2). (PDF) [file pone.0282510.s002.pdf]

Supporting information

Genetic determination of lipase and esterase activities in wheat (*Triticum aestivum L.*)

Short title: GWAS of lipase and esterase in wholegrain wheat flour

Chun Yue Wei [1], Steven Yates [2], Dan Zhu [1], Andreas Hund [3], Bruno Studer [2], Laura Nyström [1*]

[1] Laboratory of Food Biochemistry, Institute of Food, Nutrition and Health, ETH Zurich, Zurich, Switzerland

[2] Molecular Plant Breeding, Institute of Agricultural Sciences, ETH Zurich, Zurich, Switzerland

[3] Crop Science, Institute of Agricultural Sciences, ETH Zurich, Zurich, Switzerland

* Corresponding author

Email: laura.nystroem@hest.ethz.ch (LN)

S2 Table. Significant SNP markers that pass the Bonferroni corrected LOD limit. The table shows the trait, chromosome, the first and third marker positions, the marker triplet, LOD, and the variance explained (adjusted R^2).

Trait	Chromosome	First Marker Position	Third Marker Position	Triplet	LOD	Adjusted R^2
esterase_2016	1A	1.9E+08	2.0E+08	Excalibur_rep_c68921_433/IACX19890/wsnp_Ex_c10595_17291999	6.0	0.09
esterase_2016	1A	5.4E+08	5.4E+08	Ku_c8992_405/wsnp_Ex_rep_c102067_87314043/RAC875_rep_c105766_652	5.6	0.07
esterase_2016	1A	5.4E+08	5.4E+08	wsnp_Ex_rep_c102067_87313597/Tdurum_contig56158_60/RAC875_c12348_720	5.7	0.07
esterase_2016	1A	5.4E+08	5.4E+08	Tdurum_contig56158_60/RAC875_c12348_720/RFL_Contig5334_831	5.7	0.07
esterase_2016	1A	5.4E+08	5.4E+08	RAC875_c12348_720/RFL_Contig5334_831/wsnp_Ex_c28900_37982485	5.4	0.07
esterase_2016	1B	4.3E+06	4.3E+06	BS00022504_51/Excalibur_rep_c113987_164/BS00022505_51	5.5	0.08
esterase_2016	1B	4.3E+06	4.5E+06	BS00030667_51/Kukri_rep_c106834_139/RAC875_c24163_155	5.5	0.08
esterase_2016	1D	1.9E+07	1.9E+07	IAAV7510/IACX9354/D_F5XZDLF01A85DT_301	5.6	0.06
esterase_2016	2A	1.7E+07	1.7E+07	BS00011466_51/IAAV3985/RAC875_c829_1215	5.9	0.04
esterase_2016	2A	9.9E+07	1.0E+08	BS00104558_51/RAC875_rep_c119471_174/Tdurum_contig62073_376	5.7	0.07
esterase_2016	2A	6.1E+08	6.2E+08	BS00022896_51/BS00062679_51/GENE-4029_80	5.8	0.09
esterase_2016	2A	7.6E+08	7.6E+08	BobWhite_c5191_563/Excalibur_c39151_104/wsnp_Ku_c26323_36285697	5.7	0.08
esterase_2016	2A	7.6E+08	7.6E+08	Tdurum_contig66353_358/RAC875_c96399_610/IAAV3800	7.0	0.09
esterase_2016	2A	7.6E+08	7.6E+08	RAC875_c96399_610/IAAV3800/Excalibur_c4372_363	6.6	0.10
esterase_2016	2A	7.6E+08	7.6E+08	IAAV3800/Excalibur_c4372_363/Tdurum_contig53038_714	6.7	0.10
esterase_2016	2A	7.6E+08	7.6E+08	Tdurum_contig53038_684/IAAV3757/Excalibur_c31864_179	7.9	0.11
esterase_2016	2A	7.6E+08	7.6E+08	IAAV3757/Excalibur_c31864_179/IACX6480	6.4	0.09
esterase_2016	2A	7.6E+08	7.6E+08	Excalibur_c31864_179/IACX6480/Excalibur_c20478_641	7.0	0.10
esterase_2016	2A	7.6E+08	7.6E+08	IACX6480/Excalibur_c20478_641/RAC875_c16727_1826	6.2	0.10
esterase_2016	2B	4.4E+07	4.4E+07	wsnp_CAP11_rep_c8489_3665230/BS00022950_51/RAC875_rep_c79427_59	5.7	0.09
esterase_2016	2B	4.4E+07	4.6E+07	BS00022950_51/RAC875_rep_c79427_59/wsnp_Ex_c56027_58306755	5.6	0.09
esterase_2016	2B	1.0E+08	1.1E+08	BS00021675_51/Tdurum_contig7800_228/BS00071690_51	5.7	0.07
esterase_2016	2B	1.1E+08	1.1E+08	Tdurum_contig7800_228/BS00071690_51/BS00071689_51	5.7	0.07

esterase_2016	2B	1.1E+08	1.1E+08	BS00071690_51/BS00071689_51/Tdurum_contig14542_858	5.7	0.07
esterase_2016	2B	1.1E+08	1.1E+08	Kukri_c13747_464/Kukri_rep_c85389_715/BobWhite_s65298_124	5.4	0.07
esterase_2016	2B	1.1E+08	1.1E+08	Kukri_rep_c85389_715/BobWhite_s65298_124/RAC875_c35438_474	5.9	0.07
esterase_2016	2B	1.1E+08	1.1E+08	BobWhite_s65298_124/RAC875_c35438_474/BS00067975_51	5.5	0.07
esterase_2016	2B	1.1E+08	1.1E+08	RAC875_c35438_474/BS00067975_51/BS00068042_51	5.5	0.07
esterase_2016	2B	1.2E+08	1.2E+08	wsnp_Ku_c4042_7375053/Ku_c4042_576/wsnp_Ex_c269_518324	5.9	0.06
esterase_2016	2B	1.2E+08	1.2E+08	Ku_c4042_576/wsnp_Ex_c269_518324/Tdurum_contig77602_931	5.9	0.06
esterase_2016	2B	1.2E+08	1.2E+08	wsnp_Ex_c269_518324/Tdurum_contig77602_931/wsnp_Ex_c2430_4546479	6.0	0.07
esterase_2016	2B	1.2E+08	1.2E+08	Tdurum_contig77602_931/wsnp_Ex_c2430_4546479/Kukri_c11935_65	6.3	0.08
esterase_2016	2B	1.2E+08	1.2E+08	wsnp_Ex_c2430_4546479/Kukri_c11935_65/wsnp_BE497494B_Ta_2_1	6.3	0.08
esterase_2016	2B	1.2E+08	1.2E+08	Kukri_c11935_65/wsnp_BE497494B_Ta_2_1/wsnp_Ex_rep_c68704_67559626	6.3	0.08
esterase_2016	2B	1.2E+08	1.2E+08	wsnp_BE497494B_Ta_2_1/wsnp_Ex_rep_c68704_67559626/wsnp_CAP11_c3947_1866837	5.8	0.07
esterase_2016	2B	1.2E+08	1.2E+08	wsnp_Ex_rep_c68704_67559626/wsnp_CAP11_c3947_1866837/BS00003404_51	5.8	0.07
esterase_2016	2B	1.2E+08	1.2E+08	wsnp_CAP11_c3947_1866837/BS00003404_51/wsnp_CAP11_c3947_1867089	5.8	0.07
esterase_2016	2B	1.2E+08	1.2E+08	BS00003404_51/wsnp_CAP11_c3947_1867089/BS00022486_51	5.8	0.07
esterase_2016	2B	1.2E+08	1.2E+08	wsnp_CAP11_c3947_1867089/BS00022486_51/Kukri_c46621_143	5.8	0.06
esterase_2016	2B	1.2E+08	1.2E+08	BS00022486_51/Kukri_c46621_143/wsnp_Ex_c20786_29875033	5.8	0.06
esterase_2016	2B	7.6E+08	7.6E+08	RAC875_c25271_138/RAC875_rep_c112916_263/Excalibur_c10441_984	7.5	0.10
esterase_2016	2B	7.6E+08	7.6E+08	RAC875_rep_c112916_263/Excalibur_c10441_984/BobWhite_c10864_436	8.0	0.10
esterase_2016	2B	7.6E+08	7.6E+08	Excalibur_c10441_984/BobWhite_c10864_436/BS00080318_51	8.2	0.10
esterase_2016	2B	7.6E+08	7.7E+08	BobWhite_c10864_436/BS00080318_51/Kukri_c55996_63	7.7	0.10
esterase_2016	2B	7.6E+08	7.7E+08	BS00080318_51/Kukri_c55996_63/RAC875_c57353_245	6.5	0.09
esterase_2016	2B	7.7E+08	7.7E+08	Kukri_c55996_63/RAC875_c57353_245/Jagger_c7688_98	6.0	0.08
esterase_2016	2B	7.7E+08	7.7E+08	RAC875_c57353_245/Jagger_c7688_98/RAC875_c98387_145	5.9	0.09
esterase_2016	2B	7.7E+08	7.7E+08	Jagger_c7688_98/RAC875_c98387_145/Kukri_c34553_89	5.5	0.09
esterase_2016	2B	7.7E+08	7.7E+08	RAC875_c98387_145/Kukri_c34553_89/RFL_Contig3713_538	6.1	0.08

esterase_2016	2B	7.7E+08	7.7E+08	Kukri_c34553_89/RFL_Contig3713_538/BS00051965_51	5.9	0.08
esterase_2016	2B	7.7E+08	7.7E+08	RFL_Contig3713_538/BS00051965_51/Excalibur_c30744_181	6.0	0.08
esterase_2016	2B	7.7E+08	7.7E+08	BS00051965_51/Excalibur_c30744_181/RFL_Contig385_761	6.8	0.08
esterase_2016	2B	7.7E+08	7.7E+08	Excalibur_c30744_181/RFL_Contig385_761/RAC875_c22463_494	7.8	0.10
esterase_2016	2B	7.7E+08	7.7E+08	RFL_Contig385_761/RAC875_c22463_494/BobWhite_c33464_133	8.2	0.10
esterase_2016	2B	7.7E+08	7.7E+08	RAC875_c22463_494/BobWhite_c33464_133/BS00046601_51	7.6	0.10
esterase_2016	2B	7.7E+08	7.7E+08	BobWhite_c33464_133/BS00046601_51/BS00009882_51	7.3	0.10
esterase_2016	2B	7.7E+08	7.7E+08	BS00046601_51/BS00009882_51/Kukri_c24939_378	6.8	0.09
esterase_2016	2B	7.7E+08	7.7E+08	BS00009882_51/Kukri_c24939_378/BS00010438_51	6.8	0.09
esterase_2016	2B	7.7E+08	7.7E+08	Kukri_c24939_378/BS00010438_51/IACX7803	6.6	0.09
esterase_2016	2B	7.7E+08	7.7E+08	BS00010438_51/IACX7803/Excalibur_c65466_714	7.5	0.09
esterase_2016	2B	7.7E+08	7.7E+08	IACX7803/Excalibur_c65466_714/BS00069756_51	7.2	0.08
esterase_2016	2B	7.7E+08	7.7E+08	Excalibur_c65466_714/BS00069756_51/IACX5726	7.3	0.08
esterase_2016	2B	7.7E+08	7.7E+08	BS00069756_51/IACX5726/IAAV1502	7.1	0.10
esterase_2016	2B	7.7E+08	7.7E+08	IACX5726/IAAV1502/Kukri_c11040_787	7.2	0.09
esterase_2016	2B	7.7E+08	7.7E+08	IAAV1502/Kukri_c11040_787/Tdurum_contig12159_468	7.6	0.09
esterase_2016	2B	7.7E+08	7.7E+08	Kukri_c11040_787/Tdurum_contig12159_468/Tdurum_contig34075_98	7.1	0.09
esterase_2016	2B	7.7E+08	7.7E+08	Tdurum_contig12159_468/Tdurum_contig34075_98/Ex_c52711_584	6.5	0.10
esterase_2016	2D	7.7E+07	7.8E+07	IAAV2480/BobWhite_rep_c51612_864/BS00063225_51	5.9	0.09
esterase_2016	2D	4.6E+08	4.8E+08	Ku_c19185_1569/Ra_c34214_1320/Tdurum_contig13653_255	7.1	0.11
esterase_2016	2D	6.2E+08	6.2E+08	BobWhite_c36548_98/wsnp_Ex_c42970_49408712/GENE-1355_265	7.7	0.11
esterase_2016	2D	6.2E+08	6.2E+08	wsnp_Ex_c42970_49408712/GENE-1355_265/RAC875_c5673_1209	7.0	0.10
esterase_2016	2D	6.2E+08	6.2E+08	GENE-1355_265/RAC875_c5673_1209/Excalibur_rep_c102228_810	6.7	0.08
esterase_2016	2D	6.2E+08	6.2E+08	_77	5.5	0.09
esterase_2016	2D	6.2E+08	6.2E+08	Excalibur_rep_c102228_810/D_GBUVHFX01BGETX_77/Excalibur_c7051_1027	6.9	0.09
esterase_2016	2D	6.2E+08	6.3E+08	D_GBUVHFX01BGETX_77/Excalibur_c7051_1027/RAC875_c1043_258	8.3	0.12
esterase_2016	2D	6.2E+08	6.3E+08	Excalibur_c7051_1027/RAC875_c1043_258/IACX7683	6.5	0.11
esterase_2016	2D	6.3E+08	6.3E+08	RAC875_c1043_258/IACX7683/Tdurum_contig56321_232	7.3	0.12

esterase_2016	2D	6.3E+08	6.3E+08	IACX7683/Tdurum_contig56321_232/Excalibur_c91176_326	6.7	0.11
esterase_2016	3A	7.4E+08	7.4E+08	Ku_c12191_1123/Ex_c3556_2319/wsnp_Ra_c5454_9660204	5.9	0.08
esterase_2016	3B	6.2E+06	6.4E+06	Excalibur_c94546_61/BS00065248_51/Tdurum_contig34149_219	5.5	0.08
esterase_2016	3B	6.4E+06	6.6E+06	Tdurum_contig34149_219/BS00102648_51/BS00102646_51	6.7	0.09
esterase_2016	3B	6.6E+06	6.7E+06	BS00102648_51/BS00102646_51/wsnp_Ex_c2723_5047696	6.6	0.09
esterase_2016	3B	6.6E+06	6.8E+06	BS00102646_51/wsnp_Ex_c2723_5047696/Tdurum_contig14251_320	6.4	0.09
esterase_2016	3D	5.5E+08	5.5E+08	RAC875_s118882_78/IAAV5984/BS00025843_51	7.9	0.11
esterase_2016	3D	5.5E+08	5.6E+08	BS00025843_51/BobWhite_c23305_1192/Ra_c23432_639	6.9	0.10
esterase_2016	3D	5.9E+08	5.9E+08	Excalibur_c55289_304/Kukri_rep_c86903_184/wsnp_BE604885A_Ta_2_1	5.5	0.07
esterase_2016	3D	5.9E+08	5.9E+08	Kukri_rep_c86903_184/wsnp_BE604885A_Ta_2_1/GENE-2712_130	6.4	0.09
esterase_2016	3D	5.9E+08	5.9E+08	wsnp_BE604885A_Ta_2_1/GENE-2712_130/GENE-2405_141	7.5	0.10
esterase_2016	4A	6.1E+08	6.1E+08	Ra_c22675_581/RAC875_c19919_724/wsnp_Ex_c19207_28125072	5.8	0.08
esterase_2016	4A	6.1E+08	6.1E+08	RAC875_c19919_724/wsnp_Ex_c19207_28125072/Tdurum_contig97887_268	6.4	0.08
esterase_2016	4A	6.1E+08	6.1E+08	wsnp_Ex_c19207_28125072/Tdurum_contig97887_268/wsnp_Ex_c24474_33721784	7.2	0.09
esterase_2016	4A	6.1E+08	6.1E+08	Tdurum_contig97887_268/wsnp_Ex_c24474_33721784/wsnp_Ku_c3237_6024936	7.4	0.10
esterase_2016	4A	6.1E+08	6.1E+08	wsnp_Ku_c4924_8816643/IAAV7132/RAC875_c6075_214	6.5	0.08
esterase_2016	4D	1.2E+07	1.3E+07	RAC875_c6749_954/IAAV3758/Kukri_rep_c68594_530	7.8	0.10
esterase_2016	4D	1.2E+07	1.7E+07	IAAV3758/Kukri_rep_c68594_530/RAC875_c44584_162	6.3	0.10
esterase_2016	5A	3.6E+07	4.1E+07	wsnp_Ex_c356_698872/Tdurum_contig12926_687/wsnp_Ex_c28827_37918346	6.0	0.09
esterase_2016	5A	4.0E+07	4.1E+07	Tdurum_contig12926_687/wsnp_Ex_c28827_37918346/wsnp_JD_rep_c61843_39601402	6.4	0.06
esterase_2016	5A	6.9E+07	7.0E+07	BS00054690_51/wsnp_Ex_c15950_24357724/IAAV2194	6.4	0.09
esterase_2016	5A	7.0E+07	7.0E+07	wsnp_Ex_c15950_24357724/IAAV2194/RAC875_c60415_154	6.3	0.09
esterase_2016	5A	7.0E+07	7.0E+07	IAAV2194/RAC875_c60415_154/BS00064371_51	6.1	0.09
esterase_2016	5A	7.0E+07	7.4E+07	BS00064371_51/Ku_c30943_843/BS00060841_51	6.5	0.09
esterase_2016	5A	7.2E+07	7.4E+07	Ku_c30943_843/BS00060841_51/wsnp_Ku_rep_c102220_89250165	6.0	0.09
esterase_2016	5A	7.4E+07	7.5E+07	BS00060841_51/wsnp_Ku_rep_c102220_89250165/Ra_c1184_61	7.1	0.09

esterase_2016	5A	7.4E+07	7.7E+07	wsnp_Ku_rep_c102220_89250165/Ra_c1184_61/IAAV456	7.1	0.09
esterase_2016	5A	7.5E+07	7.7E+07	Ra_c1184_61/IAAV456/wsnp_Ex_rep_c66733_65077608	7.0	0.09
esterase_2016	5A	7.7E+07	7.7E+07	IAAV456/wsnp_Ex_rep_c66733_65077608/Kukri_c9885_1273	7.4	0.09
esterase_2016	5A	7.7E+07	7.8E+07	wsnp_Ex_rep_c66733_65077608/Kukri_c9885_1273/RAC875_rep_c72343_341	7.4	0.09
esterase_2016	5A	7.7E+07	7.8E+07	Kukri_c9885_1273/RAC875_rep_c72343_341/wsnp_Ra_rep_c75364_72953286	7.5	0.09
esterase_2016	5A	7.8E+07	7.8E+07	RAC875_rep_c72343_341/wsnp_Ra_rep_c75364_72953286/wsnp_Ra_rep_c75364_72953405	7.1	0.09
esterase_2016	5A	7.8E+07	7.8E+07	wsnp_Ra_rep_c75364_72953286/wsnp_Ra_rep_c75364_72953405/BobWhite_c19985_446	7.6	0.09
esterase_2016	5A	7.8E+07	7.9E+07	wsnp_Ra_rep_c75364_72953405/BobWhite_c19985_446/wsnp_Ku_c933_1913402	7.4	0.10
esterase_2016	5A	7.8E+07	8.0E+07	BobWhite_c19985_446/wsnp_Ku_c933_1913402/Kukri_c12439_876	7.1	0.10
esterase_2016	5A	7.9E+07	8.3E+07	wsnp_Ku_c933_1913402/Kukri_c12439_876/wsnp_Ex_c1981_3728899	7.1	0.10
esterase_2016	5A	8.0E+07	8.4E+07	Kukri_c12439_876/wsnp_Ex_c1981_3728899/wsnp_Ex_c29061_38130966	7.5	0.09
esterase_2016	5A	8.3E+07	8.6E+07	wsnp_Ex_c1981_3728899/wsnp_Ex_c29061_38130966/Tdurum_contig81753_70	7.4	0.08
esterase_2016	5A	8.4E+07	8.6E+07	wsnp_Ex_c29061_38130966/Tdurum_contig81753_70/Tdurum_contig81753_159	7.4	0.08
esterase_2016	5A	8.6E+07	8.6E+07	Tdurum_contig81753_70/Tdurum_contig81753_159/BobWhite_rep_c49176_485	6.9	0.08
esterase_2016	5A	8.6E+07	8.7E+07	Tdurum_contig81753_159/BobWhite_rep_c49176_485/wsnp_Ku_rep_c70261_69822367	6.4	0.09
esterase_2016	5A	8.6E+07	8.7E+07	BobWhite_rep_c49176_485/wsnp_Ku_rep_c70261_69822367/Ex_c13534_401	6.6	0.08
esterase_2016	5A	8.7E+07	9.0E+07	wsnp_Ku_rep_c70261_69822367/Ex_c13534_401/RFL_Contig4653_1217	6.6	0.08
esterase_2016	5A	8.7E+07	9.1E+07	Ex_c13534_401/RFL_Contig4653_1217/IAAV5061	7.0	0.08
esterase_2016	5A	9.0E+07	9.2E+07	RFL_Contig4653_1217/IAAV5061/wsnp_Ex_c2922_5391083	6.7	0.08

esterase_2016	5A	9.1E+07	9.2E+07	IAAV5061/wsnp_Ex_c2922_5391083/wsnp_Ra_c2228_4310870	7.1	0.08
esterase_2016	5A	9.2E+07	9.2E+07	wsnp_Ex_c2922_5391083/wsnp_Ra_c2228_4310870/RAC875_c29957_355	6.5	0.08
esterase_2016	5A	9.2E+07	9.2E+07	wsnp_Ra_c2228_4310870/RAC875_c29957_355/wsnp_JD_c8448_9444839	7.5	0.08
esterase_2016	5A	9.2E+07	9.2E+07	RAC875_c29957_355/wsnp_JD_c8448_9444839/Kukri_c40804_171	6.8	0.08
esterase_2016	5A	9.2E+07	9.4E+07	wsnp_JD_c8448_9444839/Kukri_c40804_171/wsnp_Ra_c6788_11804894	6.8	0.08
esterase_2016	5A	9.2E+07	9.7E+07	Kukri_c40804_171/wsnp_Ra_c6788_11804894/Ku_c69633_1873	7.0	0.08
esterase_2016	5A	9.4E+07	9.9E+07	wsnp_Ra_c6788_11804894/Ku_c69633_1873/wsnp_Ex_rep_c110023_92574403	7.0	0.08
esterase_2016	5A	9.7E+07	9.9E+07	Ku_c69633_1873/wsnp_Ex_rep_c110023_92574403/wsnp_Ra_c10053_16636851	7.1	0.08
esterase_2016	5A	9.9E+07	9.9E+07	wsnp_Ex_rep_c110023_92574403/wsnp_Ra_c10053_16636851/wsnp_Ku_c16812_25759885	7.1	0.08
esterase_2016	5A	9.9E+07	1.0E+08	wsnp_Ra_c10053_16636851/wsnp_Ku_c16812_25759885/wsnp_Ex_c130_259533	7.1	0.08
esterase_2016	5A	9.9E+07	1.0E+08	wsnp_Ku_c16812_25759885/wsnp_Ex_c130_259533/wsnp_Ex_c130_259006	7.0	0.08
esterase_2016	5A	1.0E+08	1.0E+08	wsnp_Ex_c130_259533/wsnp_Ex_c130_259006/wsnp_Ex_c130_258776	7.1	0.08
esterase_2016	5A	1.0E+08	1.0E+08	wsnp_Ex_c130_259006/wsnp_Ex_c130_258776/wsnp_Ku_c328_679106	6.6	0.08
esterase_2016	5A	1.0E+08	1.0E+08	wsnp_Ex_c130_258776/wsnp_Ku_c328_679106/Kukri_c92188_285	6.6	0.07
esterase_2016	5A	1.0E+08	1.0E+08	wsnp_Ku_c328_679106/Kukri_c92188_285/RAC875_rep_c114924_726	6.6	0.08
esterase_2016	5A	1.0E+08	1.0E+08	Kukri_c92188_285/RAC875_rep_c114924_726/Ex_c1344_1859	6.5	0.08
esterase_2016	5A	1.0E+08	1.1E+08	RAC875_rep_c114924_726/Ex_c1344_1859/wsnp_Ex_c6117_10704945	6.9	0.08
esterase_2016	5A	1.0E+08	1.1E+08	Ex_c1344_1859/wsnp_Ex_c6117_10704945/Kukri_c19815_106	6.9	0.08
esterase_2016	5A	1.1E+08	1.1E+08	wsnp_Ex_c6117_10704945/Kukri_c19815_106/RAC875_c20021_615	6.0	0.08
esterase_2016	5A	1.1E+08	1.1E+08	Kukri_c19815_106/RAC875_c20021_615/Ra_c28381_461	6.0	0.08
esterase_2016	5A	1.1E+08	1.1E+08	RAC875_c20021_615/Ra_c28381_461/wsnp_Ex_c18805_27686000	6.0	0.08

esterase_2016	5A	1.1E+08	1.1E+08	Ra_c28381_461/wsnp_Ex_c18805_27686000/RAC875_rep_c80358_120	6.9	0.08
esterase_2016	5A	1.1E+08	1.1E+08	wsnp_Ex_c18805_27686000/RAC875_rep_c80358_120/BS00045480_51	6.0	0.08
esterase_2016	5A	1.1E+08	1.1E+08	RAC875_rep_c80358_120/BS00045480_51/wsnp_Ra_c14112_22155312	6.0	0.08
esterase_2016	5A	1.1E+08	1.1E+08	BS00045480_51/wsnp_Ra_c14112_22155312/wsnp_Ra_c14112_22155451	6.0	0.08
esterase_2016	5A	1.1E+08	1.1E+08	wsnp_Ra_c14112_22155312/wsnp_Ra_c14112_22155451/wsnp_Ra_c18459_27525981	6.7	0.08
esterase_2016	5A	1.1E+08	1.1E+08	wsnp_Ra_c14112_22155451/wsnp_Ra_c18459_27525981/BS00045284_51	6.7	0.08
esterase_2016	5A	1.1E+08	1.1E+08	wsnp_Ra_c18459_27525981/BS00045284_51/wsnp_Ex_c15084_23263641	6.7	0.08
esterase_2016	5A	1.1E+08	1.1E+08	BS00045284_51/wsnp_Ex_c15084_23263641/wsnp_Ex_c31570_40343841	6.1	0.08
esterase_2016	5A	1.1E+08	1.2E+08	wsnp_Ex_c15084_23263641/wsnp_Ex_c31570_40343841/CAP12_rep_c5753_56	5.9	0.08
esterase_2016	5A	1.1E+08	1.2E+08	wsnp_Ex_c31570_40343841/CAP12_rep_c5753_56/CAP12_rep_c5753_98	5.6	0.08
esterase_2016	5A	1.2E+08	1.3E+08	BS00075505_51/BS00075504_51/IAAV1838	9.6	0.14
esterase_2016	5A	1.2E+08	1.4E+08	BS00075504_51/IAAV1838/RAC875_c50278_130	6.7	0.09
esterase_2016	5A	1.3E+08	1.4E+08	IAAV1838/RAC875_c50278_130/CAP7_c10833_215	5.7	0.08
esterase_2016	5A	1.4E+08	1.5E+08	RAC875_c50278_130/CAP7_c10833_215/wsnp_BE500291A_Ta_2_1	6.9	0.09
esterase_2016	5A	1.4E+08	1.5E+08	CAP7_c10833_215/wsnp_BE500291A_Ta_2_1/Kukri_rep_c69186_666	6.9	0.09
esterase_2016	5A	1.5E+08	1.6E+08	wsnp_Ex_rep_c68226_67010649/wsnp_BE444644A_Ta_2_2/wsnp_BE444644A_Ta_2_1	7.1	0.10
esterase_2016	5A	1.8E+08	2.1E+08	wsnp_Ex_c7890_13404460/Tdurum_contig42253_798/RAC875_rep_c81986_877	5.7	0.10
esterase_2016	5A	1.8E+08	2.3E+08	Tdurum_contig42253_798/RAC875_rep_c81986_877/IAAV6849	5.9	0.10
esterase_2016	5A	2.1E+08	2.3E+08	RAC875_rep_c81986_877/IAAV6849/BS00021901_51	5.8	0.09

esterase_2016	5A	4.3E+08	4.3E+08	wsnp_Ku_c5071_9050628/wsnp_Ku_c5071_9049540/BS00046529_51	5.7	0.08
esterase_2016	5A	4.3E+08	4.3E+08	wsnp_Ku_c5071_9049540/BS00046529_51/BS00039188_51	5.6	0.07
esterase_2016	5A	4.4E+08	4.5E+08	RAC875_c4033_192/BS00066569_51/Tdurum_contig48766_257	5.9	0.06
esterase_2016	5A	4.4E+08	4.5E+08	BS00066569_51/Tdurum_contig48766_257/BS00040916_51	6.0	0.04
esterase_2016	5A	5.0E+08	5.0E+08	BobWhite_rep_c64318_615/RAC875_c28819_281/BobWhite_c5457_1440	5.9	0.06
esterase_2016	5A	5.0E+08	5.1E+08	RAC875_c28819_281/BobWhite_c5457_1440/GENE-3791_463	5.9	0.07
esterase_2016	5A	5.0E+08	5.1E+08	BobWhite_c5457_1440/GENE-3791_463/Excalibur_rep_c69868_123	5.5	0.05
esterase_2016	5A	5.1E+08	5.1E+08	GENE-3791_463/Excalibur_rep_c69868_123/wsnp_Ex_c49211_53875600	5.5	0.06
esterase_2016	5A	5.4E+08	5.4E+08	BS00068178_51/Kukri_c14889_1086/Kukri_c14889_116	5.5	0.06
esterase_2016	5A	5.4E+08	5.4E+08	Kukri_c14889_1086/Kukri_c14889_116/GENE-3189_377	5.5	0.06
esterase_2016	5A	5.4E+08	5.4E+08	Kukri_c14889_116/GENE-3189_377/BS00098207_51	5.5	0.06
esterase_2016	5A	5.4E+08	5.4E+08	GENE-3189_377/BS00098207_51/RAC875_s114930_80	6.4	0.08
esterase_2016	5A	5.4E+08	5.4E+08	BS00098207_51/RAC875_s114930_80/BS00072156_51	6.4	0.08
esterase_2016	5A	5.4E+08	5.4E+08	RAC875_s114930_80/BS00072156_51/BS00072155_51	5.6	0.07
esterase_2016	5A	6.2E+08	6.2E+08	BobWhite_c14689_172/RAC875_c40928_60/wsnp_BG607308A_Ta_2_1	5.5	0.07
esterase_2016	5B	1.0E+07	1.2E+07	BS00083715_51/wsnp_Ex_c831_1625061/wsnp_Ex_c2459_4591695	5.7	0.07
esterase_2016	5B	5.6E+07	5.6E+07	RAC875_c31197_62/RAC875_c49044_445/wsnp_Ku_c7872_13484038	5.8	0.07
esterase_2016	5B	5.6E+07	5.6E+07	RAC875_c49044_445/wsnp_Ku_c7872_13484038/Ra_c19837_1031	5.8	0.07
esterase_2016	5B	5.6E+07	5.6E+07	wsnp_Ku_c7872_13484038/Ra_c19837_1031/Kukri_c43113_136	5.8	0.07
esterase_2016	5B	5.6E+07	5.7E+07	Ra_c19837_1031/Kukri_c43113_136/GENE-0782_747	5.8	0.07
esterase_2016	5B	6.5E+07	6.5E+07	Ku_c439_1308/Kukri_c439_857/RAC875_c104514_534	5.6	0.06
esterase_2016	5B	6.5E+07	6.5E+07	Kukri_c439_857/RAC875_c104514_534/wsnp_CAP8_c2693_1401039	5.5	0.06
esterase_2016	5B	6.5E+07	6.5E+07	RAC875_c104514_534/wsnp_CAP8_c2693_1401039/IAAV4252	5.5	0.06
esterase_2016	5B	7.4E+07	8.1E+07	Ku_c4349_1791/Tdurum_contig25393_218/wsnp_Ku_c11980_19464222	5.7	0.06

esterase_2016	5B	7.8E+07	8.1E+07	Tdurum_contig25393_218/wsnp_Ku_c11980_19464222/Tdurum_contig93270_1008	6.2	0.08
esterase_2016	5B	8.1E+07	8.3E+07	wsnp_Ku_c11980_19464222/Tdurum_contig93270_1008/Tdurum_contig17230_542	5.7	0.08
esterase_2016	5B	8.1E+07	8.5E+07	Tdurum_contig93270_1008/Tdurum_contig17230_542/BobWhite_c1372_133	5.8	0.09
esterase_2016	5B	1.1E+08	1.1E+08	RAC875_c96137_101/Tdurum_contig354_149/BS00064515_51	5.8	0.06
esterase_2016	5B	1.1E+08	1.2E+08	Tdurum_contig354_149/BS00064515_51/Kukri_c40388_844	5.8	0.06
esterase_2016	5B	1.1E+08	1.2E+08	BS00064515_51/Kukri_c40388_844/RFL_Contig2458_2536	9.5	0.12
esterase_2016	5B	2.9E+08	3.0E+08	Ra_c53208_433/BS00007437_51/wsnp_Ex_rep_c95506_83475785	5.7	0.08
esterase_2016	5B	3.9E+08	4.0E+08	BS00056147_51/BS00108062_51/BS00048572_51	5.6	0.08
esterase_2016	5B	4.3E+08	4.3E+08	IAAV4074/BS00028183_51/wsnp_Ex_rep_c75281_72691771	6.8	0.09
esterase_2016	5B	4.3E+08	4.3E+08	BS00028183_51/wsnp_Ex_rep_c75281_72691771/wsnp_Ex_rep_c75281_72691359	6.9	0.09
esterase_2016	5B	4.3E+08	4.3E+08	wsnp_Ex_rep_c75281_72691771/wsnp_Ex_rep_c75281_72691359/IAAV5469	6.9	0.09
esterase_2016	5B	4.3E+08	4.3E+08	wsnp_Ex_rep_c75281_72691359/IAAV5469/wsnp_Ku_c3826_7020810	7.7	0.09
esterase_2016	5B	4.3E+08	4.3E+08	IAAV5469/wsnp_Ku_c3826_7020810/BobWhite_c14223_602	6.2	0.08
esterase_2016	5B	4.3E+08	4.3E+08	RAC875_c25756_279/Kukri_c2621_610/wsnp_CAP11_c2439_1259157	6.2	0.09
esterase_2016	5B	4.4E+08	4.4E+08	Tdurum_contig10987_800/BS00070507_51/wsnp_Ku_c3869_7094615	5.7	0.08
esterase_2016	5B	4.4E+08	4.4E+08	BS00070507_51/wsnp_Ku_c3869_7094615/BS00064767_51	5.7	0.08
esterase_2016	5B	4.4E+08	4.4E+08	wsnp_Ku_c3869_7094615/BS00064767_51/wsnp_Ra_c44756_51084202	7.2	0.09
esterase_2016	5B	4.4E+08	4.4E+08	BS00064767_51/wsnp_Ra_c44756_51084202/wsnp_Ex_c6548_11355524	5.8	0.09
esterase_2016	5B	4.6E+08	4.8E+08	BS00094480_51/wsnp_Ex_c2615_4862266/Excalibur_rep_c104800_795	5.9	0.07
esterase_2016	5B	4.8E+08	4.8E+08	Excalibur_rep_c104800_795/IACX2883/wsnp_Ku_c29780_39658445	6.0	0.06
esterase_2016	5B	4.8E+08	4.8E+08	IACX2883/wsnp_Ku_c29780_39658445/wsnp_Ex_c47152_52446529	5.6	0.06

esterase_2016	5B	5.5E+08	5.5E+08	tplb0027f13_1493/wsnp_Ku_c40334_48581010/wsnp_Ex_c6695_11577150	5.8	0.08
esterase_2016	5B	5.5E+08	5.5E+08	wsnp_Ku_c40334_48581010/wsnp_Ex_c6695_11577150/Ra_c10633_2155	6.1	0.08
esterase_2016	5B	5.6E+08	5.6E+08	BS00061326_51/BS00109560_51/Kukri_rep_c68330_380	6.8	0.09
esterase_2016	5B	5.6E+08	5.6E+08	BS00109560_51/Kukri_rep_c68330_380/Excalibur_c63208_105	6.4	0.09
esterase_2016	5B	5.6E+08	5.6E+08	Kukri_rep_c68330_380/Excalibur_c63208_105/BS00068710_51	5.8	0.09
esterase_2016	5D	7.3E+07	7.4E+07	RAC875_rep_c110059_409/RFL_Contig2368_1958/RAC875_c5518_1401	5.7	0.07
esterase_2016	5D	7.4E+07	8.5E+07	RAC875_c5518_1401/RAC875_c51455_182/GENE-3606_315	9.6	0.15
esterase_2016	5D	8.0E+07	9.4E+07	RAC875_c51455_182/GENE-3606_315/wsnp_Ra_c17541_26430903	7.2	0.10
esterase_2016	5D	8.5E+07	1.1E+08	GENE-3606_315/wsnp_Ra_c17541_26430903/IACX846	9.3	0.13
esterase_2016	5D	9.4E+07	1.1E+08	wsnp_Ra_c17541_26430903/IACX846/wsnp_Ex_rep_c67164_65655648	7.7	0.11
esterase_2016	5D	2.6E+08	2.8E+08	Ra_c53868_364/wsnp_Ex_c22726_31932096/IAAV1427	5.6	0.08
esterase_2016	5D	5.4E+08	5.4E+08	BobWhite_c38741_143/Excalibur_rep_c107626_110/BobWhite_c38741_108	5.5	0.06
esterase_2016	5D	5.5E+08	5.5E+08	Ra_c2279_730/Kukri_c14692_75/D_GCE8AKX02IXEFJ_281	7.1	0.10
esterase_2016	5D	5.5E+08	5.5E+08	Kukri_c14692_75/D_GCE8AKX02IXEFJ_281/BS00022688_51	6.9	0.10
esterase_2016	5D	5.5E+08	5.5E+08	RAC875_c3672_705/IAAV7104/Jagger_c8037_96	5.7	0.09
esterase_2016	5D	5.5E+08	5.6E+08	Jagger_c8037_96/RAC875_c703_2551/BS00032035_51	6.0	0.08
esterase_2016	5D	5.6E+08	5.6E+08	RAC875_c703_2551/BS00032035_51/RAC875_c12181_1166	6.0	0.07
esterase_2016	5D	5.6E+08	5.6E+08	BS00032035_51/RAC875_c12181_1166/BobWhite_c20748_123	6.0	0.07
esterase_2016	5D	5.6E+08	5.6E+08	BobWhite_c20748_123/BS00079156_51/RAC875_c16419_585	5.8	0.08
esterase_2016	6A	9.1E+07	9.2E+07	IAAV612/tplb0028p11_1104/Tdurum_contig15238_972	5.8	0.08
esterase_2016	6A	1.1E+08	1.2E+08	IACX14305/wsnp_Ra_c16745_25482384/GENE-2561_69	6.9	0.09
esterase_2016	6A	5.0E+08	5.0E+08	Kukri_rep_c106170_174/BS00066623_51/wsnp_Ex_c34545_42832894	5.7	0.09
esterase_2016	6A	6.1E+08	6.1E+08	Excalibur_c25390_2483/Tdurum_contig30953_142/Excalibur_c16590_1536	5.8	0.07
esterase_2016	6A	6.1E+08	6.1E+08	Tdurum_contig42418_2618/tplb0055o21_1994/IAAV6777	6.3	0.08
esterase_2016	6A	6.1E+08	6.1E+08	tplb0055o21_1994/IAAV6777/BS00067558_51	6.3	0.07
esterase_2016	6A	6.1E+08	6.1E+08	RAC875_c27781_591/BS00099879_51/wsnp_Ex_c21129_30256617	8.2	0.10

esterase_2016	6A	6.1E+08	6.1E+08	BS00099879_51/wsnp_Ex_c21129_30256617/BobWhite_c40602_313	8.1	0.10
esterase_2016	6A	6.1E+08	6.1E+08	wsnp_Ex_c21129_30256617/BobWhite_c40602_313/RAC875_c13595_438	8.1	0.10
esterase_2016	6A	6.1E+08	6.1E+08	BobWhite_c40602_313/RAC875_c13595_438/tplb0037a05_913	7.9	0.10
esterase_2016	6A	6.1E+08	6.1E+08	RAC875_c13595_438/tplb0037a05_913/Excalibur_rep_c103232_355	7.3	0.10
esterase_2016	6A	6.1E+08	6.1E+08	tplb0037a05_913/Excalibur_rep_c103232_355/wsnp_Ra_c21546_30949373	6.2	0.09
esterase_2016	6A	6.1E+08	6.1E+08	IAAV5595/Excalibur_rep_c68035_128/wsnp_Ex_c749_1472258	5.9	0.09
esterase_2016	6A	6.1E+08	6.1E+08	Excalibur_rep_c68035_128/wsnp_Ex_c749_1472258/tplb0031m24_341	5.9	0.09
esterase_2016	6A	6.1E+08	6.1E+08	wsnp_Ex_c749_1472258/tplb0031m24_341/BS00094893_51	7.5	0.09
esterase_2016	6A	6.1E+08	6.1E+08	tplb0031m24_341/BS00094893_51/wsnp_Ku_c1468_2912654	7.4	0.09
esterase_2016	6A	6.1E+08	6.1E+08	BS00094893_51/wsnp_Ku_c1468_2912654/RFL_Contig5262_1500	7.3	0.10
esterase_2016	6A	6.1E+08	6.1E+08	wsnp_Ku_c1468_2912654/RFL_Contig5262_1500/Excalibur_rep_c90454_251	7.6	0.10
esterase_2016	6A	6.1E+08	6.1E+08	RFL_Contig5262_1500/Excalibur_rep_c90454_251/BS00009600_51	7.7	0.10
esterase_2016	6A	6.1E+08	6.1E+08	BS00009600_51/BS00046261_51/JD_c6831_221	5.9	0.08
esterase_2016	6A	6.1E+08	6.1E+08	wsnp_Ex_c26771_35998816/Tdurum_contig29607_413/wsnp_Ex_c26771_35998435	5.9	0.08
esterase_2016	6A	6.1E+08	6.1E+08	Excalibur_rep_c98042_438/wsnp_Ex_rep_c69054_67959458/Ex_c69054_723	5.6	0.07
esterase_2016	6A	6.1E+08	6.1E+08	wsnp_Ex_rep_c69054_67959458/Ex_c69054_723/Excalibur_rep_c69054_795	5.6	0.07
esterase_2016	6A	6.1E+08	6.1E+08	Ex_c69054_723/Excalibur_rep_c69054_795/IACX5481	5.6	0.07
esterase_2016	6A	6.1E+08	6.1E+08	Excalibur_rep_c69054_795/IACX5481/RFL_Contig4069_2628	7.4	0.10
esterase_2016	6A	6.1E+08	6.1E+08	IACX5481/RFL_Contig4069_2628/BobWhite_c10832_1131	9.4	0.13
esterase_2016	6B	3.2E+07	3.4E+07	BS00029386_51/wsnp_BE404947B_Ta_2_12/BS00067133_51	8.4	0.12
esterase_2016	6B	1.6E+08	1.6E+08	Tdurum_contig6121_907/Tdurum_contig6121_947/Excalibur_c63243_434	6.2	0.09
esterase_2016	6B	1.6E+08	1.6E+08	Tdurum_contig6121_947/Excalibur_c63243_434/wsnp_Ex_rep_c66552_64838102	5.9	0.09

esterase_2016	6B	1.6E+08	1.6E+08	wsnp_Ex_rep_c66552_64838102/Kukri_rep_c68102_99/wsnp_Ex_rep_c66552_64837613	5.5	0.06
esterase_2016	6B	1.6E+08	1.6E+08	Kukri_rep_c68102_99/wsnp_Ex_rep_c66552_64837613/GENE-3659_104	5.9	0.08
esterase_2016	6B	2.1E+08	2.1E+08	BobWhite_c17750_568/Kukri_c66290_127/Excalibur_c3810_186	5.5	0.06
esterase_2016	6B	2.1E+08	2.1E+08	Kukri_c66290_127/Excalibur_c3810_186/wsnp_Ex_c43809_50018316	5.5	0.06
esterase_2016	6B	2.1E+08	2.1E+08	RAC875_c10122_113/Ra_c77985_260/wsnp_Ra_c26091_35652620	6.4	0.09
esterase_2016	6B	2.1E+08	2.2E+08	Ra_c77985_260/wsnp_Ra_c26091_35652620/IAAV9195	6.2	0.10
esterase_2016	6B	2.1E+08	2.2E+08	wsnp_Ra_c26091_35652620/IAAV9195/BS00041505_51	6.0	0.10
esterase_2016	6B	6.8E+08	6.8E+08	tplb0021a17_853/wsnp_Ku_c43368_50890819/Tdurum_contig32579_121	5.6	0.07
esterase_2016	6B	6.8E+08	6.8E+08	wsnp_Ex_c1383_2651887/Ku_c32100_105/RAC875_c12907_515	6.5	0.08
esterase_2016	6B	6.8E+08	6.8E+08	RAC875_c12907_515/wsnp_Ex_c34123_42489621/RAC875_rep_c108460_542	6.1	0.08
esterase_2016	6B	6.8E+08	6.8E+08	wsnp_Ex_c34123_42489621/RAC875_rep_c108460_542/BS00067983_51	6.3	0.09
esterase_2016	6B	6.8E+08	6.8E+08	RAC875_rep_c108460_542/BS00067983_51/BS00067388_51	7.1	0.11
esterase_2016	6B	7.0E+08	7.0E+08	BS00067417_51/GENE-3863_132/RAC875_rep_c113188_81	5.7	0.11
esterase_2016	6B	7.1E+08	7.1E+08	wsnp_Ex_c3025_5587183/Tdurum_contig43335_1521/RAC875_c31299_1302	5.4	0.07
esterase_2016	6B	7.1E+08	7.1E+08	RAC875_c23654_214/Jagger_c1231_85/Kukri_c338_330	7.6	0.08
esterase_2016	6D	7.6E+07	7.9E+07	GENE-4045_141/Tdurum_contig54967_722/RAC875_c52566_644	5.6	0.09
esterase_2016	6D	7.9E+07	8.7E+07	RAC875_c52566_644/BS00064893_51/RAC875_c65227_99	7.0	0.11
esterase_2016	6D	4.6E+08	4.6E+08	GENE-3871_337/GENE-3852_1100/Excalibur_c24688_407	6.2	0.09
esterase_2016	6D	4.6E+08	4.6E+08	GENE-3852_1100/Excalibur_c24688_407/GENE-3862_178	6.2	0.09
esterase_2016	6D	4.6E+08	4.6E+08	Excalibur_c24688_407/GENE-3862_178/GENE-3862_349	6.2	0.09
esterase_2016	6D	4.6E+08	4.6E+08	GENE-3862_178/GENE-3862_349/Kukri_c15205_882	5.8	0.08
esterase_2016	6D	4.6E+08	4.6E+08	GENE-3862_349/Kukri_c15205_882/tplb0046a04_902	6.0	0.09
esterase_2016	6D	4.6E+08	4.6E+08	D_GDEEGVY02GMZ4L_337/Excalibur_c50656_660/RAC875_c38615_143	5.6	0.08
esterase_2016	6D	4.6E+08	4.6E+08	wsnp_Ex_c14691_22765150/IAAV3944/RAC875_c13595_669	7.5	0.11
esterase_2016	6D	4.6E+08	4.6E+08	IAAV3944/RAC875_c13595_669/RAC875_c63262_67	7.5	0.11

esterase_2016	6D	4.6E+08	4.6E+08	RAC875_c13595_669/RAC875_c63262_67/D_GA8KES401DISTP_65	8.1	0.11
esterase_2016	6D	4.6E+08	4.6E+08	RAC875_c63262_67/D_GA8KES401DISTP_65/CAP8_c2210_103	8.3	0.11
esterase_2016	6D	4.6E+08	4.6E+08	D_GA8KES401DISTP_65/CAP8_c2210_103/wsnp_Ex_rep_c69248_68171036	7.1	0.11
esterase_2016	6D	4.6E+08	4.6E+08	CAP8_c2210_103/wsnp_Ex_rep_c69248_68171036/Ku_c107983_831	6.6	0.10
esterase_2016	6D	4.6E+08	4.6E+08	Kukri_rep_c107983_607/Ex_c5168_849/Excalibur_c5168_512	6.2	0.09
esterase_2016	6D	4.6E+08	4.6E+08	Ex_c5168_849/Excalibur_c5168_512/RAC875_c82385_209	6.1	0.09
esterase_2016	6D	4.6E+08	4.6E+08	Excalibur_c5168_512/RAC875_c82385_209/Kukri_rep_c68091_971	6.2	0.09
esterase_2016	6D	4.7E+08	4.7E+08	BS00065028_51/Excalibur_c22876_1040/RFL_Contig5722_537	5.6	0.08
esterase_2016	6D	4.7E+08	4.7E+08	Excalibur_c22876_1040/RFL_Contig5722_537/BobWhite_c12261_130	5.9	0.07
esterase_2016	7A	4.8E+07	4.9E+07	RAC875_c52560_123/wsnp_CAP12_c3056_1439567/RAC875_rep_c109924_289	6.0	0.09
esterase_2016	7A	1.3E+08	1.3E+08	IACX1777/BS00056927_51/BS00022435_51	5.4	0.07
esterase_2016	7A	1.3E+08	1.3E+08	BS00056927_51/BS00022435_51/BS00094744_51	6.0	0.08
esterase_2016	7A	1.3E+08	1.3E+08	BS00022435_51/BS00094744_51/BS00022834_51	6.0	0.08
esterase_2016	7A	6.1E+08	6.1E+08	BS00003494_51/BS00092631_51/wsnp_Ex_c1146_2200823	6.1	0.08
esterase_2016	7B	1.3E+06	3.5E+06	Kukri_c21384_1475/Excalibur_c8486_419/BS00022127_51	5.6	0.06
esterase_2016	7B	8.3E+07	8.4E+07	BS00024215_51/Excalibur_c59263_96/CAP7_c10566_170	5.5	0.08
esterase_2016	7B	7.1E+08	7.1E+08	Excalibur_c1205_188/BobWhite_c4729_243/BobWhite_c6690_965	6.4	0.10
esterase_2016	7B	7.1E+08	7.1E+08	BobWhite_c6690_965/BobWhite_rep_c55035_124/GENE-4528_455	5.9	0.09
esterase_2016	7B	7.1E+08	7.1E+08	IAAV4582/BS00066404_51/RAC875_rep_c69334_132	6.0	0.08
esterase_2016	7B	7.1E+08	7.1E+08	BS00066404_51/RAC875_rep_c69334_132/wsnp_Ex_c25821_35085420	5.6	0.08
esterase_2016	7D	2.0E+07	2.1E+07	Kukri_c9104_1124/Excalibur_c16355_712/RAC875_c6280_292	9.3	0.13
esterase_2016	7D	2.0E+07	2.2E+07	Excalibur_c16355_712/RAC875_c6280_292/IACX8322	9.8	0.13
lipase_2015	3B	6.8E+08	6.9E+08	BS00093891_51/BS00048754_51/BS00083391_51	5.7	0.10
lipase_2016	1A	5.4E+08	5.4E+08	wsnp_Ex_rep_c102067_87313597/Tdurum_contig56158_60/RAC875_c12348_720	5.6	0.07
lipase_2016	1A	5.4E+08	5.5E+08	Tdurum_contig15015_883/IACX14/BS00036104_51	6.0	0.09
lipase_2016	2B	6.5E+07	6.5E+07	RAC875_c61801_299/RAC875_c15229_108/BobWhite_c47608_273	5.9	0.08

lipase_2016	2B	6.5E+07	6.5E+07	RAC875_c15229_108/BobWhite_c47608_273/RAC875_c34516_316	6.1	0.08
lipase_2016	3A	7.4E+08	7.4E+08	Ku_c12191_1123/Ex_c3556_2319/wsnp_Ra_c5454_9660204	6.9	0.09
lipase_2016	4A	7.1E+08	7.1E+08	wsnp_CAP7_c254_138937/wsnp_CAP7_c254_139077/RAC875_c17185_6 66	6.3	0.07
lipase_2016	4A	7.1E+08	7.1E+08	wsnp_CAP7_c254_139077/RAC875_c17185_666/CAP7_c254_486	6.9	0.07
lipase_2016	4A	7.1E+08	7.1E+08	RAC875_c17185_666/CAP7_c254_486/RFL_Contig3841_3051	6.2	0.06
lipase_2016	4A	7.1E+08	7.1E+08	CAP7_c254_486/RFL_Contig3841_3051/Tdurum_contig75819_1471	6.1	0.08
lipase_2016	4A	7.1E+08	7.1E+08	RFL_Contig3841_2433/RFL_Contig3841_2409/Tdurum_contig45738_670	5.7	0.07
lipase_2016	4A	7.1E+08	7.1E+08	RFL_Contig3841_2409/Tdurum_contig45738_670/BS00039811_51	6.1	0.07
lipase_2016	4A	7.1E+08	7.1E+08	Tdurum_contig45738_670/BS00039811_51/BS00093289_51	7.0	0.09
lipase_2016	4A	7.1E+08	7.1E+08	BS00039811_51/BS00093289_51/BS00075049_51	7.0	0.09
lipase_2016	4A	7.1E+08	7.1E+08	BS00093289_51/BS00075049_51/wsnp_Ku_c20783_30448706	7.2	0.09
lipase_2016	4A	7.1E+08	7.1E+08	BS00075049_51/wsnp_Ku_c20783_30448706/Kukri_c20783_1016	5.9	0.09
lipase_2016	4A	7.1E+08	7.1E+08	Kukri_c20783_1016/BobWhite_c20306_88/RAC875_rep_c114991_62	6.6	0.07
lipase_2016	4B	5.4E+05	6.4E+05	Tdurum_contig11733_825/BS00056495_51/Tdurum_contig81460_347	6.8	0.08
lipase_2016	4B	6.2E+05	1.0E+06	BS00056495_51/Tdurum_contig81460_347/RAC875_c86104_111	6.2	0.08
lipase_2016	4B	6.4E+05	1.3E+06	Tdurum_contig81460_347/RAC875_c86104_111/wsnp_Ku_c12399_2003 7334	6.0	0.07
lipase_2016	5B	3.8E+08	3.9E+08	Tdurum_contig84745_267/BS00022652_51/wsnp_Ex_c1630_3105100	5.7	0.07
lipase_2016	5B	3.9E+08	3.9E+08	wsnp_Ex_c1630_3105100/BS00064333_51/BS00056147_51	5.7	0.09
lipase_2016	5B	3.9E+08	4.0E+08	BS00056147_51/BS00108062_51/BS00048572_51	5.6	0.08
lipase_2016	5B	5.6E+08	5.6E+08	BS00061326_51/BS00109560_51/Kukri_rep_c68330_380	5.6	0.06
lipase_2016	5B	5.6E+08	5.6E+08	Kukri_rep_c68330_380/Excalibur_c63208_105/BS00068710_51	5.6	0.09
lipase_2016	6A	1.1E+07	1.2E+07	Kukri_c25244_199/BS00098857_51/RFL_Contig1622_1259	5.8	0.08
lipase_2016	6A	1.2E+07	1.2E+07	BS00098857_51/RFL_Contig1622_1259/RFL_Contig1622_923	5.6	0.08
lipase_2016	6A	8.6E+07	8.6E+07	wsnp_Ex_rep_c67878_66584488/Kukri_c9595_242/wsnp_Ex_c35465_43 610634	5.5	0.06
lipase_2016	6A	8.6E+07	8.7E+07	BS00084846_51/BS00039145_51/RFL_Contig2605_672	5.6	0.06
lipase_2016	6A	8.7E+07	8.8E+07	BS00039145_51/RFL_Contig2605_672/Kukri_c7794_1247	5.8	0.06

lipase_2016	6A	8.7E+07	8.8E+07	RFL_Contig2605_672/Kukri_c7794_1247/Kukri_c3075_391	5.8	0.06
lipase_2016	6A	8.8E+07	8.8E+07	Kukri_c7794_1247/Kukri_c3075_391/wsnp_Ex_rep_c102845_87922204	5.8	0.06
lipase_2016	6A	8.8E+07	8.8E+07	Kukri_c3075_391/wsnp_Ex_rep_c102845_87922204/IAAV583	5.7	0.06
lipase_2016	6A	8.8E+07	8.8E+07	wsnp_Ex_rep_c102845_87922204/IAAV583/RAC875_c767_4007	5.8	0.06
lipase_2016	6A	8.8E+07	8.8E+07	IAAV583/RAC875_c767_4007/BS00004377_51	5.8	0.07
lipase_2016	6A	8.8E+07	8.8E+07	RAC875_c767_4007/BS00004377_51/IACX6453	5.8	0.07
lipase_2016	6A	8.8E+07	8.9E+07	BS00004377_51/IACX6453/Kukri_c16463_584	5.7	0.07
lipase_2016	6A	8.8E+07	9.0E+07	IACX6453/Kukri_c16463_584/BS00092676_51	5.7	0.07
lipase_2016	6A	8.9E+07	9.1E+07	Kukri_c16463_584/BS00092676_51/IAAV612	5.7	0.07
lipase_2016	6A	9.0E+07	9.2E+07	BS00092676_51/IAAV612/tplb0028p11_1104	6.5	0.07
lipase_2016	6A	9.1E+07	9.2E+07	IAAV612/tplb0028p11_1104/Tdurum_contig15238_972	6.0	0.09
lipase_2016	7D	2.0E+07	2.1E+07	Kukri_c9104_1124/Excalibur_c16355_712/RAC875_c6280_292	6.0	0.09
lipase_2016	7D	2.0E+07	2.2E+07	Excalibur_c16355_712/RAC875_c6280_292/IACX8322	6.1	0.09
